# *Bacteroidota* inhibit microglia clearance of amyloid-beta and promote plaque deposition in Alzheimer’s disease mouse models

**DOI:** 10.1038/s41467-024-47683-w

**Published:** 2024-05-08

**Authors:** Caroline Wasén, Leah C. Beauchamp, Julia Vincentini, Shuqi Li, Danielle S. LeServe, Christian Gauthier, Juliana R. Lopes, Thais G. Moreira, Millicent N. Ekwudo, Zhuoran Yin, Patrick da Silva, Rajesh K. Krishnan, Oleg Butovsky, Laura M. Cox, Howard L. Weiner

**Affiliations:** 1 Ann Romney Center for Neurologic Diseases, Brigham & Women’s Hospital, Harvard Medical School, Boston, MA USA; 2 Department of Life Sciences, Chalmers University of Technology, Gothenburg, Sweden; 3 Department of Rheumatology and Inflammation Research, Sahlgrenska Academy, University of Gothenburg, Gothenburg, Sweden; 4 Department of Ophthalmology, Massachusetts Eye and Ear, Harvard Medical School, Boston, MA USA

**Keywords:** Neuroimmunology, Microbiome, Neurodegeneration

## Abstract

The gut microbiota and microglia play critical roles in Alzheimer’s disease (AD), and elevated *Bacteroides* is correlated with cerebrospinal fluid amyloid-β (Aβ) and tau levels in AD. We hypothesize that *Bacteroides* contributes to AD by modulating microglia. Here we show that administering *Bacteroides fragilis* to APP/PS1-21 mice increases Aβ plaques in females, modulates cortical amyloid processing gene expression, and down regulates phagocytosis and protein degradation microglial gene expression. We further show that administering *Bacteroides fragilis* to aged wild-type male and female mice suppresses microglial uptake of Aβ1-42 injected into the hippocampus. Depleting murine *Bacteroidota* with metronidazole decreases amyloid load in aged 5xFAD mice, and activates microglial pathways related to phagocytosis, cytokine signaling, and lysosomal degradation. Taken together, our study demonstrates that members of the *Bacteroidota* phylum contribute to AD pathogenesis by suppressing microglia phagocytic function, which leads to impaired Aβ clearance and accumulation of amyloid plaques.

## Abstract (web summary)

The gut microbiota and microglia play critical roles in Alzheimer’s disease (AD). Here, the authors show that Bacteroides fragilis contributes to AD pathogenesis in mouse models by suppressing immune-mediated microglial clearance of amyloid beta.

## Introduction

The gut microbiome is altered in Alzheimer’s disease (AD) and studies have found that depleting or transplanting the gut microbiota has a significant impact on amyloid pathology in models of AD. Germ-free APP/PS1 and 5xFAD mice have lower amyloid-β (Aβ) levels in the brain^1–3^ and colonizing germ-free APP/PS1 mice with the gut microbiome of conventionally raised APP/PS1 mice increased Aβ levels^2^. Furthermore, transplanting gut microbiota from aged 5xFAD mice led to reduced hippocampal neurogenesis and memory impairment in wild type (WT) mice^4^, and colonizing ADLP^APT^ mice with the microbiota from WT mice reduced both Aβ and tau^5^. The age of the donor mouse may play a role in this effect, as 5xFAD mice who received the microbiota of aged (1-year old) WT mice developed more amyloid plaques compared to mice receiving the microbiota from young (1-month-old) donors. While these studies demonstrate that changes in the gut microbiota can contribute to AD, the role of specific bacterial species in the development of AD is currently not well established.

The gut microbiota plays an important role in modulating microglia functions, and may play a role in immune clearance of amyloid from the brain in models of AD. Changes in microglia morphology, activation status, and transcriptional profile have been observed in APP/PS1 mice whose microbiota was depleted with antibiotics^6^, and these changes were reversed by transplanting the microbiome from APP/PS1 donor mice^7^, demonstrating that microglia are an important link between the gut microbiota and amyloid pathology. The gut microbiota also supports homeostatic microglial function, as germ-free or antibiotic treated WT mice had impaired microglia maturation and function, which could be partially restored by orally administered bacteria-derived short-chain fatty acids^8^.

Modification of the gut microbiota by depletion or transplantation of gut microbiota between animals demonstrates that there is an important connection between the gut and AD. A limited number of reports have investigated which specific bacteria that are related to aging and AD pathology^9^, but the mechanistic link is still unknown. Increased levels of *Bacteroides* has been reported in the APP/PS1^1^ and Tg2576^10^ AD animal models, and *Bacteroides* was associated with higher levels of Aβ in female mice^10^. AD patients have a higher abundance of *Bacteroides* compared to age-matched controls^11,12^. Furthermore, increased levels of *Bacteroides* has been reported in aging^13^, and elderly individuals with higher abundance of gut *Bacteroides* have a higher mortality^14^. *Bacteroides fragilis*, the most extensively studied species of the *Bacteroides* genus, can have a dual role in health and disease^15^. Some strains have a beneficial role in inflammatory diseases and depression by inducing regulatory T cells (Tregs) or by producing gamma-aminobutyric acid (GABA)^16^. Other *B. fragilis* strains have a detrimental role as infectious agents^15^; and may have pathogenicity factors such as the fragilysin toxin, which is a matrix metalloproteinase that can disrupt the integrity of the gut barrier and promote inflammatory bowel disease and colon cancer. Several members of the *Bacteroidota* family have been associated with AD in humans, including *B. fragilis, B. vulgatus*, and *Alistipes*. We chose to focus on the “Type” strain of the *Bacteroidota* phylum, *B. fragilis*, because we have already established that it increases Aβ plaques^10^, which has now been confirmed by an independent study^17^. Thus, *B. fragilis* is an ideal model organism to investigate immune mechanisms by which the microbiota affects AD.

In the present study, we orally administered the non-toxigenic type-strain of *B. fragilis* (ATCC 25285) to APP/PS1-21 mice at the age when they begin to develop amyloid plaques. We found that mice receiving *B. fragilis* had increased plaques and decreased microglial gene expression associated with phagocytosis and protein degradation. *B. fragilis* also suppressed the in vivo microglia uptake of Aβ peptides in aged mice. Furthermore, we found that the Gram-negative anaerobe-targeting antibiotic metronidazole (MTZ) reduced plaques and modulated microglia gene expression related to phagocytosis when given to aged 5xFAD mice. Taken together, our studies identify immune mechanisms by which specific bacteria can contribute to AD pathogenesis.

## Results

### *Bacteroides fragilis* increases plaque burden and expression of amyloid processing genes in cortex

**Fig. 1:**
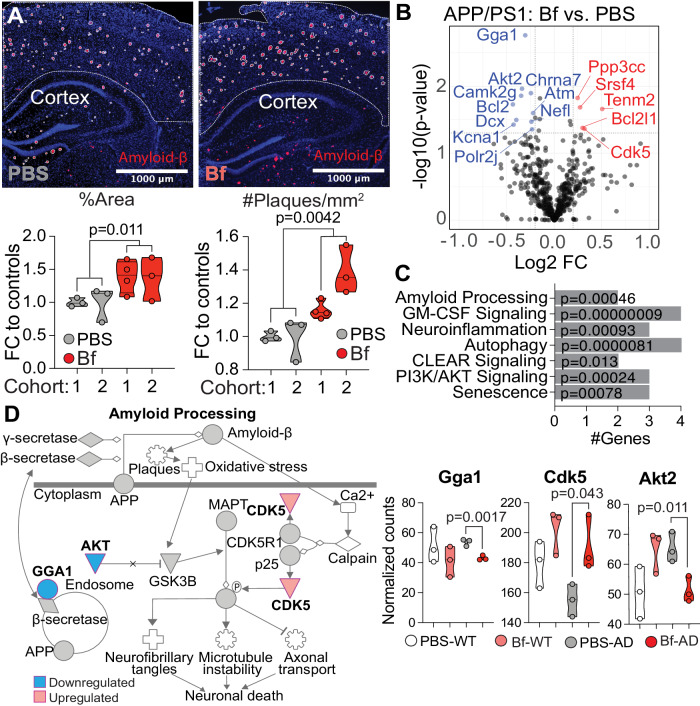
*Bacteroides fragilis* increases plaque burden and expression of amyloid processing genes in cortex. APP/PS1 mice were treated with *Bacteroides fragilis* (Bf) between 2.6–5.0 months of age. **A** Amyloid plaque burden was assessed by immunohistofluorescence of the cortex. The graph represents the fold change (FC) of plaques in mice treated with Bf vs. controls treated with PBS. Plaque burden is defined as the percent of the cortex area that were covered with plaques and the number of plaques per square millimeter in the cortex. The data is pooled from two independent experiments, PBS: *n* = 6 mice/group, Bf: *n* = 7. Three mice from cohort 2 belonging to the PBS group was excluded from the analysis because the tissue was damaged during cutting. Histologic findings from cohort 1 are a modified representation of data originally published in Cox et al., Scientific reports, 2019, under the Creative Commons CC BY license https://www.nature.com/articles/s41598-019-54187-x#rightslink. The *p*-values were calculated with a two-sided Student’s *t* test. **B**–**D** The cortical tissue from cohort 1 was analyzed for transcriptional changes with the NanoString Neuropathology panel, the statistical test was two-sided. **B** Volcano plot of differentially expressed genes between APP/PS1 mice treated with Bf or PBS. **C** Pathways identified by Ingenuity Pathway Analysis (IPA) in APP/PS1 mice treated with Bf vs. PBS, genes with FC > ±0.2 and a *p*-value < 0.05 were included in the analysis. **D** Schematic figure of the amyloid processing pathway (adaption of IPA-generated graph) and graphs showing the differentially expressed genes in the pathway, the *p*-values were generated with NSolver Advanced Analysis. *n* = 3 mice/group. Violin plots represent min, max, interquartile range and median, the dots represent mice. Source data are provided as a Source Data file.

We have previously observed correlations between the abundance of *Bacteroides* and Aβ levels in the brain of female mice^10^. To investigate if *B. fragilis* accelerates Aβ deposition, we administered *B. fragilis* to two independent cohorts of female APP/PS1 and WT mice from 2.6 to 5.0 months of age^10^ (Supplementary Table 1), starting at the stage of early plaque formation. We found that *B. fragilis* increased Aβ plaques in the cortex as measured both by a 38% increase in percent area (2-tailed t-test, *t* = 3.1, df = 11, *p* = 0.011) and 26% increase in plaque number (2-tailed t-test, *t* = 3.6, df = 11, *p* = 0.0042, Fig. 1A). No changes were observed in the hippocampus (Supplementary Fig. 1A). There were no significant changes in amyloid burden of male mice in cortex or the hippocampus (Supplementary Fig. 2A, B). We investigated the effect of *B. fragilis* on cortical tissue gene expression using the NanoString Neuropathology panel (Fig. 1B). Ingenuity pathway analysis (IPA) (Fig. 1C) of APP/PS1-Bf vs. APP/PS1-PBS indicated that the differentially expressed genes (DEGs, 10 downregulated and 5 upregulated genes) were involved in amyloid processing (*p* = 0.00046, Fig. 1D), including reduced expression of *Gga1* (mean normalized counts of 43.1 vs. 53.7, *p* = 0.0017) that regulates endosomal trafficking of β-secretase from the cell membrane^18^, reduced expression of *Akt2* (51.2 vs. 65.3, *p* = 0.011) that inhibits GSK3B which participates in Aβ production and tau hyperphosphorylation^19^, and higher expression of Cdk5 (191 vs. 155, *p* = 0.043) which contributes to AD pathology by phosphorylating both the amyloid precursor protein (APP) and Tau^20^. Other pathways affected by *B. fragilis* administration indicated immune signaling, including neuroinflammation (IPA-analysis, *p* = 0.00093), and GM-CSF signaling (IPA-analysis, *p* = 0.00024, Fig. 1C, Supplementary Fig. 3A). Changes in immune signaling was also observed in the periphery of APP/PS1 mice, where CD4^+^ T cells had a 56.9% reduced production of GM-CSF (1-way ANOVA, *t* = 2.68, df = 29, adjusted *p* = 0.024, Supplementary Fig. 3B), but no difference in IL-10, IL-17 or IFN-g (Supplementary Fig. 3C–E). Cortical tissue also had altered gene expression related to senescence (IPA-analysis, *p* = 0.00078) and clearance (IPA-analysis, *p* = 0.013, Fig. 1C).

### *Bacteroides fragilis* downregulates microglia genes involved in plaque clearance

**Fig. 2:**
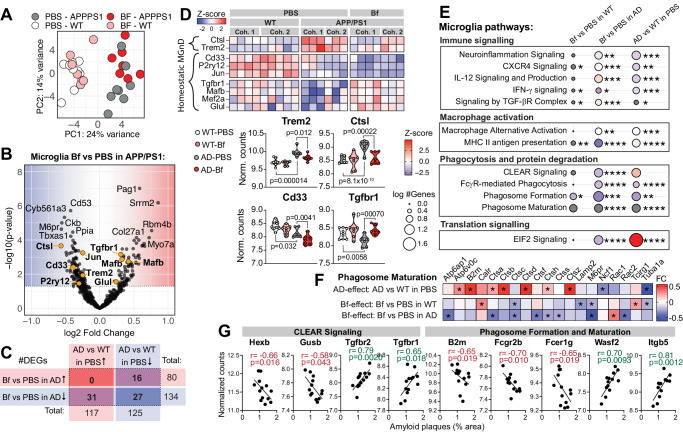
*Bacteroides fragilis* downregulates microglia genes involved in plaque clearance. Microglia were isolated from APP/PS1 (Alzheimer’s disease, AD) and wild type (WT) female mice treated with *B. fragilis* (Bf) between 2.6–5.0 months of age and analyzed by RNA sequencing. Data from two independent experiments were pooled in the analysis (cohort 1 and 2). Transcripts with an average count of 100 (scaled by size factors) across all samples were included in the analysis. *P*-values and fold changes were calculated with two-sided Wald test using the DESeq2 package in R. **A** Principal component (PC) plot. **B** Volcano plot showing *p*-value and fold change (FC) of genes in APP/PS1 mice treated with Bf vs. PBS (controls). **C** Table showing the number of up- and downregulated genes (*p*-value < 0.05) in microglia from APP/PS1 mice treated with Bf vs. PBS and in PBS treated mice of APP/PS1 vs. WT genotype. **D** Heatmap showing differentially expressed genes (DEGs, Bf vs. PBS in APP/PS1 mice) that are typically up- (MGnD) or downregulated (homeostatic) in microglia with a neurodegenerative phenotype compared to homeostatic microglia. Selected genes are also shown in boxplots representing the normalized counts. **E** Altered pathways identified by Ingenuity Pathway Analysis (IPA), for genes with FC > ±0.2 and a *p*-value < 0.05. Circles are shaded according to the z-score indicating activation (red) or inhibition (blue) or no presumed direction (gray). The size of the circle corresponds to the number of genes in the pathway, *p < 0.05, **p < 0.01, ***p < 0.001, ****p < 0.0001, *p*-values were generated by IPA. **F** Heatmap of the FC of genes in the Phagosome Maturation Pathway, * *p* < 0.05. WT-PBS: *n* = 9 mice/group, WT-Bf: *n* = 7, APP/PS1-PBS: *n* = 8, APP/PS1-Bf: *n* = 7. **G** Spearman correlation between genes from pathways related to phagocytosis and protein degradation and the percent area of amyloid plaques in the cortex. Violin plots represent min, max, interquartile range and median, the dots represent mice. Source data are provided as a Source Data file.

Microglia play an important role in brain homeostasis by clearing amyloid and preventing plaque formation^21^. Because our analysis of cortical tissue from *B. fragilis* treated mice indicated immune signaling and changes in gene expression related to clearance, we next performed RNA sequencing on sorted microglia from the same mice. A principal component analysis revealed distinct clusters between WT and APP/PS1 mice, and a partial separation between APP/PS1 mice treated with *B*. fragilis and PBS (control) (Fig. 2A). *B. fragilis* upregulated 80 genes and downregulated 134 genes in female APP/PS1 mice (DESeq2’s Wald test, *p*-value < 0.05, Fig. 2B). Of these genes, 74 genes were differentially expressed in both APP/PS1-PBS mice vs. APP/PS1-*B. fragilis* (bacterial effect) and in WT-PBS vs. APP/PS1-PBS (AD effect) (Supplementary Fig. 4A, B). Surprisingly, all the DEGs of *B. fragilis* treated mice were either downregulated or regulated in the opposite direction (Fig. 2C), suggesting that *B. fragilis* suppresses the physiologic microglia response to Aβ in APP/PS1 mice.

In AD, microglia change from homeostatic to a neurodegenerative (MGnD) phenotype and several upregulated MGnD genes facilitate Aβ clearance^21^. We found that treatment with *B. fragilis* suppressed MGnD genes in APP/PS1 mice including a reduction in *Trem2* expression (means of normalized counts: 9.83 vs. 9.98, Wald test, *p* = 0.012, Fig. 2D), which is important for clearance of plaque by microglia^22^ and a reduction in Cathepsin L (*Ctsl*, means: 0.61 vs. 9.01, Wald test, *p* > 0.001) which is a degrading enzyme in the microglia lysosome^23^. We also found that *Cd9* was downregulated, which is associated with the disease-associated-microglia signature^24^ and the phagocytosis marker macrosialin (*Cd68*) (Supplementary Fig. 4B). Four homeostatic genes that were downregulated in APP/PS1 were upregulated to the same level as WT mice by *B. fragilis* (Fig. 2D), including the TGF-β receptor (*Tgfbr1*, means: 8.43 vs. 8.13, Wald test, *p* > 0.001). Three genes, *CD33* (means: 7.89 vs. 8.15, Wald test, *p* = 0.0041), *P2ry12* (means: 2122 vs. 3223, Wald test, *p* = 0.035), and *Jun* (means: 2522 vs. 4004, Wald test, *p* = 0.00057), were downregulated in APP/PS1 mice and further downregulated by *B. fragilis*. Downregulation of *P2ry12* and *Jun* were also confirmed by NanoString of sorted microglia in cohort 2 (*p *= 0.053 and *p *= 0.026, respectively), together with *Ctnnb1*, *Lamp1*, *Fcgr1*, *Csf3r*, *Psen1*, *Stat3*, *Ndufa2*, *Fos* and *Hsp1a/b* (Supplementary Fig. 4C, D).

Ingenuity Pathway Analysis (IPA) of microglia DEGs indicated that pathways related to macrophage alternative activation (IPA z-score = 0, *p* = 0.0079) and MHC-II antigen presentation (IPA z-score = −3.00, *p* > 0.001) were affected by the treatment with *B. fragilis* in APP/PS1 mice (Fig. 2E). Also, cytokine signaling, including Il-12 (IPA z-score = 1.00, *p* < 0.001), TGF-β (IPA z-score = 0.45, *p* < 0.001) and IFN-γ (IPA z-score = −2, *p* = 0.012) signaling was affected. Interestingly, the IPA Z-score indicated a suppression of phagocytosis and protein degradation in APP/PS1 mice treated with *B. fragilis* (Fig. 2E), including FcγR-mediated phagocytosis (IPA z-score = −1.41, *p* < 0.001), phagosome formation (IPA z-score = −2.67, *p* < 0.001), phagosome maturation (IPA z-score = NA, *p* < 0.001) and coordinated-lysosomal-expression-and-regulation (CLEAR) signaling (IPA z-score = −1.51, *p* < 0.001). Downregulated genes related to phagosome maturation included several protein-cleaving cathepsins, acidifying H + -ATPases and other lysosomal membrane proteins (Fig. 2F). Furthermore, some of the phagosome/lysosome related DEGs also correlated to amyloid plaque levels in the cortex of the mice (Fig. 2G).

To investigate if changes in the microglia phenotype was related to the microglia’s ability to migrate to plaques, we stained brain tissue from *B. fragilis* treated females for the microglia marker Iba-1. While *B. fragilis* treated mice tended to have a higher number of microglia associated with plaques that likely reflected their increased plaque burden (30.9% higher in mice treated with B. fragilis, 2-tailed t-test, *t* = 2.22, df = 4, *p* = 0.09), there was no difference in number of microglia per total plaque area (0.0039 microglia/plaque area unit in *B. fragilis* vs. 0.0041 in PBS) or the percentage of the microglia that were associated with plaques (23.0% in *B. fragilis* vs. 25.8% in PBS, Supplementary Fig. 1B).

Male mice did not have altered plaque burden in response to *B. fragilis* (3.68% in B. fragilis vs. 2.96% in PBS, 2-tailed t-test, *t* = 0.85, df = 10, *p* = 0.41), but we did observe an upregulation of 108 genes and a downregulation of 131 genes (DESeq2’s Wald test, *p*-value < 0.05) in microglia (Supplementary Fig. 2C). As opposed to our findings in female mice, *B. fragilis* did not modulate MGnD/homeostatic genes *Trem2, Cstl, CD33* and *Tgfbr1* in male mice (Supplementary Fig. 2D). We identified 33 genes were regulated by *B. fragilis* in the opposite direction of gene expression altered in APP/PS1 vs. WT mice, which is smaller than we observed in female mice (74 genes) (Supplementary Fig. 2E). According to the pathway analysis, phagosome maturation was affected by *B. fragilis* treatment (Supplementary Fig. 2F), although to a lesser extent than in the females (IPA z-score −2.7 in females and −0.58 in males, *p* = 0.023). Taken together, these data suggest that *B. fragilis* suppresses genes involved in immune-mediated clearance of Aβ, with greater effects in female mice, which may partially account for sex-specific plaque accumulation.

### *Bacteroides fragilis* inhibits Aβ uptake by microglia

**Fig. 3:**
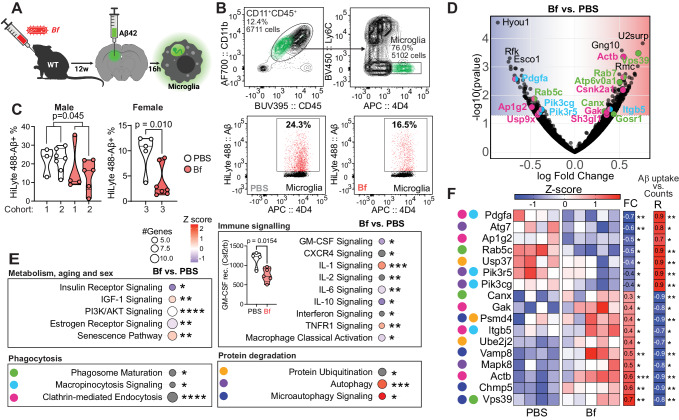
*Bacteroides fragilis* inhibits Aβ uptake by microglia. **A** 8–12-month-old male (data pooled from two independent experiments) and female wildtype (WT) mice were given weekly gavages of *B. fragilis* (Bf) for 2 months. Fluorescently labeled Amyloid β (Aβ)−42 was injected into the hippocampus and microglia sorted 16 h later. Created with BioRender.com. **B** Gating strategy for isolation of microglia from the brain and assessment of Aβ uptake. **C** Microglial uptake of Aβ in mice treated with Bf. *p*-values were calculated using a two-sided Mann–Whitney U test. **D** Volcano plot of differentially expressed genes in microglia isolated from Bf treated mice cohort 3) and controls and analyzed by RNA sequencing. *P*-values and fold changes were calculated with two-sided Wald test using the DESeq2 package in R. **E** Pathways altered by Bf identified by Ingenuity Pathway Analysis (IPA), including genes with a FC > ±0.2 and a *p*-value < 0.05. Circles are shaded according to the z-score indicating activation (red) or inhibition (blue) or no presumed direction (gray). The size of the circle corresponds to the number of genes in the pathway, **F** Expression of microglial genes involved in phagocytosis and protein degradation pathways indicated by the colored circle: phagocytosis (green), macropinocytosis (light blue), clathrin-mediated endocytosis (pink), protein ubiquitination (yellow), autophagy (purple), and microautophagy (dark blue).  Individual replicates are shown, as well as fold change (FC) per group and significant diffferenced detected by DESeq2. Spearman R of amyloid uptake vs. normalised counts and significance *p*-values are shown in the right column. Male-PBS: *n* = 10 mice/group, Male-Bf: *n* = 10, Female-PBS: *n* = 5, Female-Bf: *n* = 7. Violin plots represent min, max, interquartile range and median, the dots represent mice. *p < 0.05, **p < 0.01, ***p < 0.001, ****p < 0.0001. Source data are provided as a Source Data file.

We next investigated whether *B. fragilis* inhibited microglia clearance of Aβ in vivo. For this purpose, we treated three independent cohorts of aged WT mice (Supplementary Table 2) and two cohorts of APP/PS1 mice (Supplementary Table 2) with *B. fragilis*, PBS or control bacteria for 2 months. We then then injected fluorescently labeled Aβ-42 into the hippocampus and measured the ability of microglia to phagocytose Aβ-42 by flow cytometry 16 h later (Fig. 3A, B). For these experiments we chose to use aged mice, because age is a major risk factor for AD and aging may lead to differences in both barrier permeability and microglia function. We utilized two controls, a PBS (vehicle) control and 3 strains of *Erysipelotrichaceae* (E3) to serve as a non-*B. fragilis* bacterial control (Supplementary Table 2), which were selected based on our previous findings that *Erysipelotrichaceae* were associated with lower levels of Aβ40/42^10^. We found that treatment with *B. fragilis* led to reduced Aβ uptake vs. PBS in both female (2.25% vs. 10.9% Aβ-42^+^ microglia, 2-tailed Mann–Whitney test, U = 2.00, *p* = 0.010) and male (13.75% vs. 23.5%, 2-tailed Mann–Whitney test, U = 23.5, exact *p* = 0.045, Fig. 3C) WT mice. We did not see an effect of *B. fragilis* in APP/PS1 mice on clearing exogenous FITC-Aβ42, likely due to the high levels of endogenous amyloid load at initiation of injection (Supplementary Fig. 5B). Furthermore, microglia from WT mice treated with E3 tended to increase Aβ uptake vs. PBS (4.21% in E3 vs. 2.46% in PBS, 2-tailed Mann–Whitney test, U = 9.00, *p* = 0.053, Supplementary Fig. 5A), demonstrating that the addition of any bacteria does not result in the same suppressive effect as *B. fragilis*.

To identify microglia functions associated with impaired phagocytosis due to *B*. fragilis, we performed RNA sequencing on WT female microglia from our Aβ injections. Administering *B. fragilis* modulated DEGs involved in macrophage classical activation (IPA z-score = −0.45, *p*-value = 0.034), immune signaling including GM-CSF signaling (IPA z-score = −1.00, *p*-value = 0.012) with reduced expression of the GM-CSF receptor (Csf2rb, Fig. 3D, E). *B. fragilis* also affected pathways involved in phagocytosis and protein degradation including phagosome maturation (IPA z-score = NA, *p*-value = 0.019), macropinocytosis signaling (IPA z-score = NA, *p*-value = 0.016) and clathrin-mediated endocytosis (IPA z-score = NA, *p*-value < 0.001) (Fig. 2E, F). Genes from these pathways also correlated with the Aβ-42 uptake by the microglia (Fig. 3F), associating their expression with microglia function. Among these genes were *Pik3r5* and *Pik3cg*, which are activated by the GM-CSF receptor (and other receptors) and control macropinocitosis signaling and autophagy. *Pik3r5* and *Pik3cg* had a positive correlation with Aβ-42 uptake (Spearman R = 0.92 and 0.88, *p* = 0.0013 and 0.031, respectively) and were downregulated by treatment with *B. fragilis* (DESeq2’s Wwald test, FC = −0.37 and −0.40, *p* = 0.031 and *p* = 0.040, respectively). Other genes, such as the endolysosomal trafficking protein *Vps39* was negatively associated with Aβ-42 uptake (Spearman R = −0.83, *p* = 0.0083) and were upregulated by *B. fragilis* (FC = 0.71, *p* = 0.00032). Taken together, these studies demonstrate that *B. fragilis* directly impairs Aβ clearance by microglia and identifies several pathways involved in Aβ phagocytosis that are modulated by *B. fragilis*.

### Metronidazole increases insulin degrading enzyme in the cortex and reduces amyloid plaques

MTZ is an antibiotic that targets Gram-negative anaerobes including *B. fragilis*. Given that mice colonized with *B. fragilis* had increased amyloid plaques, we asked whether treatment with MTZ would also reduce the plaque burden in older mice when plaques had already developed. We used 5xFAD mice that carry the same human transgenes as APP/PS1 mice and have additional mutations associated with familial AD. We supplemented the drinking water of aged female 5xFAD mice with 0.2 mg/mL MTZ during the first week and then 0.04 mg/mL MTZ for 9 weeks starting at 9 months of age (Supplementary Table 3). Analysis of the most abundant bacterial phyla demonstrated that MTZ treatment reduced the relative abundance of *Bacteroidota* (median relative abundance 40.4% at 28d vs. 58.8% before MTZ, 2-tailed paired Wilcoxon test, exact *p* = 0.020) and increased *Firmicutes* (median relative abundance 52.6% at 28d vs. 61.6% before MTZ, 2-tailed paired Wilcoxon test, exact *p* = 0.020) in feces after 28 days, while controls had no differences in these phyla over time (Supplementary Fig. 6A, B). Both MTZ treated mice and controls had higher levels of *Campilobacteriota* 49 days after the treatment started, and no difference in *Desulfobacteriota* was observed at any timepoint. We also analyzed which bacteria differed between MTZ treated mice and control in at least two timepoints after the start of MTZ treatment, and/or in the ileum or cecum at the time of sacrifice. Although we did not detect *B. fragilis* in any of the samples, we found reduced abundance of closely related members of the *Bacteroidota* phylum. The most abundant amplicon sequence variants that were reduced after MTZ treatment belonged to the *Muribaculaceae* family (median relative abundance in feces was 2.22% after 7d of MTZ treatment vs. 3.87% in controls, LEfSe analysis, LDA-score −4.05, *p* = 0.043; median relative abundance in cecum 1.09% after 70d of MTZ treatment vs. 4.92% in controls, LEfSe analysis, LDA-score = −3.95, *p* = 0.034), which is the dominant *Bacteroidota* member in mice^25^. In addition, the mice had lower levels of other anaerobic bacteria belonging to the *Odoribacter* genus, the *Ruminococcaceae* family, the genus *Butyricicoccus*, *Lactospiraceae* NK4A136 group, *Lachnoclostridium* and *Desulfovibrio* and the class *Coriobacteriia*. Of note, there was an increase of *Oscillospiraceae*, which have been reported to not be depleted in response to MTZ^26^ (Supplementary Fig. 6C).

**Fig. 4:**
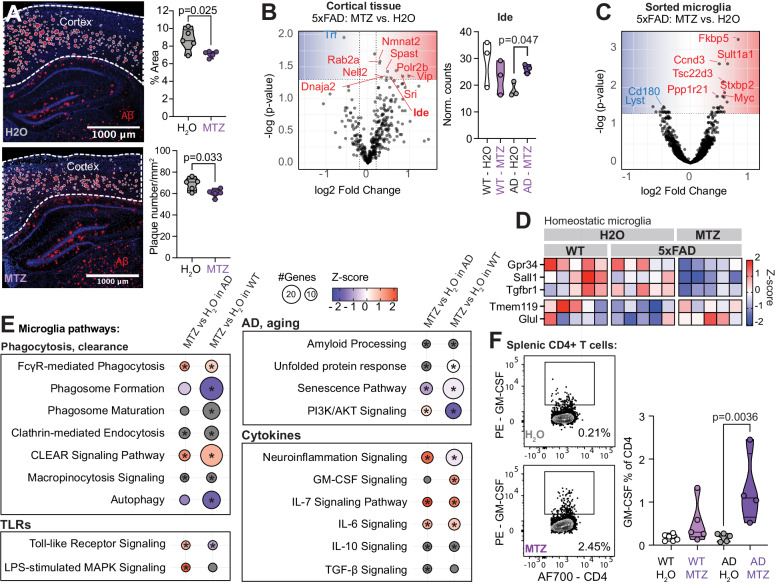
Metronidazole reduces amyloid plaques and modulates central and peripheral immunity. Female 5xFAD and wild type (WT) mice were treated with metronidazole (MTZ) in their drinking water between 9 and 12 months of age. Amyloid plaques were assessed with histology, cortical gene expression with the NanoString neuropathology panel and microglia gene expression with RNA sequencing. **A** The amyloid-β (Aβ) plaque burden in cortex. Plaque burden was defined as the percent of the cortex area that were covered with plaques and the number of plaques per square millimeter in the cortex. The *p*-values were calculated with a two-sided Student’s *t* test, *n* = 5 mice/group. **B** Volcano plot of differentially expressed genes in the cortical tissue of 5xFAD-MTZ mice vs. 5xFAD-H_2_0 mice and the normalized counts of Ide mRNA (coding for Insulin degrading enzyme), the *p*-value was generated with Nsolver Advanced Analysis (two-sided test), *n* = 3 mice/group. **C** Volcano plot showing the adjusted two-sided *p*-value and fold change (FC) of microglia genes modulated by MTZ in 5xFAD mice. **D** Heatmap of homeostatic microglia gene signature with an unadjusted two-sided *p*-value of <0.05 (5xFAD-H_2_O vs. 5xFAD-MTZ), *n* = 5 mice/group. **E** Microglia pathways altered by MTZ in 5xFAD mice identified by Ingenuity Pathway Analysis (IPA). Genes with a FC > ±0.2 and a two-sided unadjusted *p*-value < 0.05 were included in the analysis. Circles are shaded according to the z-score indicating activation (red) or inhibition (blue) or no presumed direction (gray). *p-value < 0.05, *n* = 5 mice/group. **F** Frequency of GM-CSF + T cells, the T cells were isolated from the spleen and stimulated for 3 h with PMA/ionomycin. One outlier was removed from the AD-MTZ group with the Grubb’s test in GraphPad Prism. The *p*-values were calculated with one-way ANOVA, *n* = 5 mice/group. Violin plots represent min, max, interquartile range and median, the dots represent individual mice. Source data are provided as a Source Data file.

As shown in Fig. 4A, we found that MTZ treatment reduced Aβ plaque percent area (7.10% area in MTZ vs. 8.70% in H_2_O, 2-tailed t-test, *t* = 2.00, df = 8, *p* = 0.025) and number (60.4 plaques/mm^2^ vs. 68.6, 2-tailed t-test, *t* = 2.58, df = 8, *p* = 0.033) in the cortex. No difference was observed in the hippocampus (Supplementary Fig. 7). Transcriptional analysis of cortical tissue revealed that MTZ also modulated cortical gene expression in WT and 5xFAD mice (Fig. 4B). In both WT and 5xFAD mice, the majority of DEGs modulated by MTZ were upregulated (WT: 66 up, 15 down, 5xFAD: 15 up, 3 down, *p* < 0.05). MTZ had a larger effect on gene expression in WT mice compared to 5xFAD and there was no overlap between DEGs (MTZ vs. H_2_O) in WT and 5xFAD animals (Supplementary Fig. 8A–C). In 5xFAD mice, we found that MTZ upregulated insulin degrading enzyme (*Ide*, *p* = 0.047), which is known to degrade Aβ^27^ (Fig. 4B). Taken together, these results suggest that depletion of *Bacteroidota* reduce plaque burden, that MTZ has differential effects in WT and 5xFAD cortical gene expression and that MTZ can modulate genes involved in amyloid degradation in 5XFAD mice.

In sorted microglia, MTZ modulated 41 genes in 5xFAD mice (DESeq2’s Wald test, *p* < 0.05, Fig. 4C, Supplementary Fig. 9A, B), of which 7 genes were also modulated by MTZ in WT mice. We identified 5 homeostatic microglia DEGs, three genes (*Gpr34, Sall1* and *Tgfbr1*) that were downregulated in AD mice treated with MTZ (DESeq’s Wald test, *p* = 0.0015, *p* = 0.011 and *p* = 0.00080, respectively) and two genes (*Tmem119* and *Glul*) were upregulated (*p* = 0.017 and *p* = 0.0070, Fig. 4D). Ingenuity pathway analysis showed that MTZ affected several pathways related to AD and aging in both 5xFAD and WT mice including amyloid processing, senescence, phagocytosis, protein clearance, TLR activation, and cytokine signaling (Fig. 4E). Interestingly, we found that the GM-CSF pathway was activated in WT mice (IPA z-score = 1.34, *p* = 0.022) and found that splenic T cells produced more GM-CSF in 5xFAD mice (0.18% GM-CSF^+^ cells in MTZ vs. 1.29% in H_2_O, 1-way ANOVA, *t* = 3.88, df = 17, *p* = 0.0036, Fig. 4F). The FcγR-mediated phagocytosis (MTZ-AD vs. H_2_O-AD: IPA z-score = 0.63, *p* < 0.001; MTZ-WT vs. H_2_O-WT: IPA z-score = 1.34, *p*-value = 0.0067) and the CLEAR signaling pathways (MTZ-AD vs. H_2_O-AD: IPA z-score = 1.00, *p* < 0.001; MTZ-WT vs. H_2_O-WT: IPA z-score = 1.41, *p*-value = 0.029) were predicted to be activated by MTZ treatment, suggesting that microglia clearance of Aβ played a role in the lower plaque burden observed in 5xFAD mice.

## Discussion

Recent studies indicate that the gut microbiota contributes to AD, but additional data is needed to define the role of specific bacteria and the mechanisms by which they act. Our study reports that *Bacteroides* contributes to AD pathology by suppressing immune-mediated Aβ clearance. Studies have reported elevated levels of *Bacteroides* in AD subjects^11,12,28^ and high levels of *Bacteroides* have been linked to an increased risk of mortality in elderly individuals^14^, supporting a role for *Bacteroides* in age-related diseases. We have previously reported that administration of *B. fragilis* resulted in higher amyloid plaque burden in the cortex of APP/PS1 mice^10^ which was confirmed with an additional cohort of mice described in this study. We observed differences in amyloid pathology primarily in the cortex, which is the location where plaque deposition starts in the APP/PS1-21 model^29^. Another recent study reported that administration of *B. fragilis* also resulted in higher levels of aggregated Aβ in the cortex of Thy1-C/EBPβ mice^17^, and attributed the effect to poly-unsaturated fatty acid metabolism by *B. fragilis* leading to increased levels of 12-hydroxy-heptadecatrienoic acid and Prostaglandin E2 in cortical tissue. In the Thy1-C/EBPβ model, depletion of microglia attenuated the effect of *B. fragilis* metabolites^17^, but whether *B. fragilis-*mediated microglia modifications lead to pathology in Aβ models has not been previously described.

Here, we deeply phenotype microglia from mice treated with *B. fragilis* with both RNA sequencing and an in vivo functional assay to demonstrate that *B. fragilis* inhibits microglia clearance of Aβ. We found *B. fragilis* induced similar transcriptional changes in microglia in APP/PS1 mice with elevated Aβ in the cortex and in WT mice when Aβ had been injected into the hippocampus, suggesting that there are no regional differences in microglia response to *B. fragilis*. Because the role of microglia in AD changes throughout the course of disease^3^, we chose to analyze microglia from relatively young APP/PS1 mice. At these earlier stages of AD, microglia are believed to have an important protective role by clearing amyloid from the brain. However, in the advanced stages of disease, microglia take on a pro-inflammatory and neurotoxic role^21^. Transcriptional analysis of microglia from APP/PS1 mice demonstrated that *B. fragilis* reduced expression of proteins that participate in receptor-mediated phagocytosis, phagosome formation and maturation, including proteins involved in the degradation of engulfed proteins. Of note, TLR4-dependent lipopolysaccharide (LPS) stimulation of microglia has been reported to suppress autophagy leading to the impaired capacity to degrade phagocytized Aβ^30^. It has also been shown that blocking TLR2 signaling can promote lysosomal Aβ-degradation by microglia which is also connected to metabolic changes favoring glycolysis^31^. Thus, it is possible that one of the mechanisms by which *B. fragilis* impairs Aβ clearance is by disrupting autophagy and lysosomal function in microglia.

We also assessed microglial function in vivo and found that *B. fragilis* impaired microglial uptake of injected Aβ peptides in aged WT mice, which is consistent with our transcriptional data. In addition to providing a functional connection between *B. fragilis* and microglia clearance, this model complements our previous results by exposing the microglia to amyloid starting at an advanced age. This is important because the gut and brain barriers may become more permeable for bacterial products with age, and transgenic models in which mice develop plaques at a very young age may not be ideal models of the gut-brain connection in age-related diseases^32,33^.

RNA sequencing of the microglia from *B. fragilis* treated WT mice challenged with Aβ hippocampal injection identified several pathways related to phagocytosis and protein degradation that were affected by *B. fragilis*, including the maturation of the phagosome. Furthermore, genes from phagocytosis- and degradation-related pathways correlated to the uptake of amyloid peptides, supporting their relevance for Aβ uptake. In AD, microglia change from a homeostatic to a neurodegenerative phenotype^21^ named MGnD, which are involved in Aβ clearance and repair. We found that treatment with *B. fragilis* blocked transcriptional changes associated with the MGnD phenotype in microglia, including Trem2, which was associated with a higher plaque burden in APP/PS1 mice. Indeed, several of the MGnD associated genes support plaque-clearing functions in microglia^34^, and suppression of these genes may impair the microglia’s ability to respond to the amyloid peptides.

One potential mechanism by which the gut microbiota may affect AD pathology is peripheral immune modulation that influences microglia function. Others have hypothesized that *B. fragilis* contributes to AD by inducing inflammation^17,35^. However, we did not observe increased inflammation in WT or APP/PS1 mice, as measured by production of IFNγ, IL-17, and GM-CSF in splenic CD4^+^ T cells or by microglia transcription and function. Rather, *B. fragilis* suppressed the production of GM-CSF by splenic CD4^+^ T cells in APP/PS1 mice, suggesting reduced pro-inflammatory stimulation from T cells. Of note, APP/PS1-PBS mice had higher GM-CSF compared to WT-PBS, suggesting that elevated GM-CSF may be a normal compensatory response to Aβ pathology in this model, and the *B. fragilis-*mediated suppression of GM-CSF may contribute to disease. In support of this theory, previous work has demonstrated that GM-CSF promotes microglia proliferation, induces an ameboid morphology associated with microglia activation and induce phagocytic capacity of cultured microglia^36^. GM-CSF also stimulated phagocytosis by microglia when injected into the brain^36^. Furthermore, administration of GM-CSF to APP/PS1 mice reduced amyloid plaques in vivo^37^ and reduced amyloid plaques in brain slices when added directly to the tissue^38^, demonstrating that GM-CSF enhances the ability to clear Aβ. Furthermore, GM-CSF has been recognized as a potential therapy in AD and is currently in phase II clinical trial^39^. Of note, others have reported an immune suppressive function of *B. fragilis* including animal models of inflammatory disease^16^, consistent with the immunosuppressive effect we see in this model.

Another mechanism by which the gut microbiota affects neurologic disease is the secretion of metabolites and peptides. Studies have shown that bacterial metabolites that originate from bacteria in the gut can be found in cerebrospinal fluid^40^ and brain tissue from humans^41^, and bacterial outer-membrane vesicles has been found in the brains of mice^42^. Thus, bacterial products originating from the gut may interact directly with cells in the brain. Prior studies report that administering short-chain fatty acids (SCFA) to germ-free or specific pathogen-free (conventionally raised) APP-PS1-21 Jucker mice promoted plaques production^43^, which was linked to changes in microglial transcriptional profiles and reduced Aβ phagocytosis. Furthermore, acetate, the predominant microbial SCFA, increases amyloid plaque levels in germ-free 5xFAD mice^44^. Of note, *Bacteroides* is known to produce the short chain fatty acids, acetate, succinate, and propionate, thus modulating *Bacteroides* may affect SCFA^45^. Furthermore, intraperitoneal injection of *B. fragilis* derived metabolites has been shown to influence AD pathology in the Thy1-C/EBPβ model.^17^ This study also found that *B. fragilis* increased the escape latency in the Morris water maze, suggesting that *B. fragilis* may contribute to memory impairments. Mechanisms by which *B. fragilis* contributes to AD via secreted components warrants further study.

Based on our *B. fragilis* data, we hypothesized that administering an antibiotic that targeted *Bacteroides* and related bacteria in the phylum *Bacteroidota* would also reduce amyloid plaques. Because studies report that *Bacteroides* is increased in aging and because administering MTZ to young mice may have limited effects due to age-related microbiota composition, we chose to treat older animals^1,10,46^. We administered MTZ to 5xFAD mice at a dose of 0.2 mg/mL during the first week and then 0.04 mg/mL for 9 weeks starting at 9 months of age, which is well after Aβ plaque deposition, to model a treatment paradigm. We found that MTZ reduced plaque burden in cortex and increased splenic GM-CSF, and increased microglial genes involved in phagocytosis. Consistent with our hypothesis, we found that MTZ decreased *Bacteroidota*, specifically members *Muribaculaceae* and *Odoribacter*. Thus, our data suggests that a reduction Gram-negative anaerobes closely related to *Bacteroides *in the *Bacteroidota* family may have a protective effect in AD. Of note, we also reported that a few other, less abundant, species were also reduced in relative abundance by MTZ. These included butyrate producing *Firmicutes*, but since butyrate producers has been previously suggested to support gut barrier function^47^ and microglia function^48^, these bacteria are unlikely to contribute to Aβ pathology. Likewise, a reduction in *Desulfovibrio*, an acetate producer, could potentially contribute to plaques as acetate has been shown to increase amyloid load in 5xFAD^44^. Lastly, while *Coriobacteriia*, it has been associated with cognitive decline in patients with hypertension^49^, little is known about whether it modulates microglia. In summary, the results from our MTZ investigations support our initial finding that *Bacteroidota* contribute to amyloid accumulation.

Other studies using early-life treatment regimens have not found a protective effect of MTZ on Aβ pathology^50^. Dodiya et al. reported that early-life MTZ administered to male APP/PS1-21 mice between 2 weeks – 2 months of age did not impact the cortical plaque burden, whereas a combination of MTZ, kanamycin gentamicin, colistin and vancomycin significantly reduced plaques^50^. Antibiotics can have variable effects on the gut microbiota, depending on the host endogenous microbiota and may be dependent on the animal facility, the strain of mouse used, the age, and the sex. One study has reported that a short-term antibiotic treatment of APP/PS1 mice at 3 months of age did not change the plaque burden at 5 months^51^. Another study has found that a plaques are reduced in GF and antibiotic treated 5xFAD mice, using a mixture of vancomycin, cefoxitin, gentamycin, and MTZ (1 mg/mL)^3^. This reduction in plaques was only associated with increased microglial phagocytosis GF mice, not antibiotic treated mice. Furthermore, plaque reduction and microglia phagocytosis changes were only observed at 4 months in 5xFAD mice, and not seen after a cumulative 10 months of microbiota depletion^3^. Because MTZ can cross the blood brain barrier and long-term high dose treatment may become neurotoxic^52^. Thus, our shorter treatment with lower dose MTZ (0.2 mg/mL) may account for the protective effect we found in aged mice. Furthermore, the microbiota changes throughout aging, and several independent studies have reported an age-dependent increase in *Bacteroidota* in humans^13,14^ and the APP/PS1, 5xFAD, and Tg2576 models of AD^1,10,46^. These differences in the bacteria composition in young vs. aged animal models could potentially explain why MTZ alone given to young mice have limited effects on amyloid pathology in mice^50^.

In addition, studies reported an age-dependent decrease in *Erysipelotrichaceae* family member *Faecalibaculum rodentium* in 5xFAD and Tg2576 mice^10,46^, and found a negative correlation between *Faecalibaculum* and levels of Aβ^10^. Interestingly, antibiotic treatment of AD subjects with rifaximin increased the *Erysiplelotrichaceae* family member *Erysipeloclostridium* and reduced serum levels of phosphorylated tau^53^. In our study, we found that administering a combination of *Erysipelotrichaceae*, including *F. rodentium, I. valens* and *D. newyorkensis* tended to increase the in vivo microglia clearance of amyloid peptides injected into the brains of aged WT mice. Taken together, these results suggest that *Erysiplelotrichaceae* might play an important role amplifying the protective functions of microglia against amyloid pathology in AD.

**Fig. 5:**
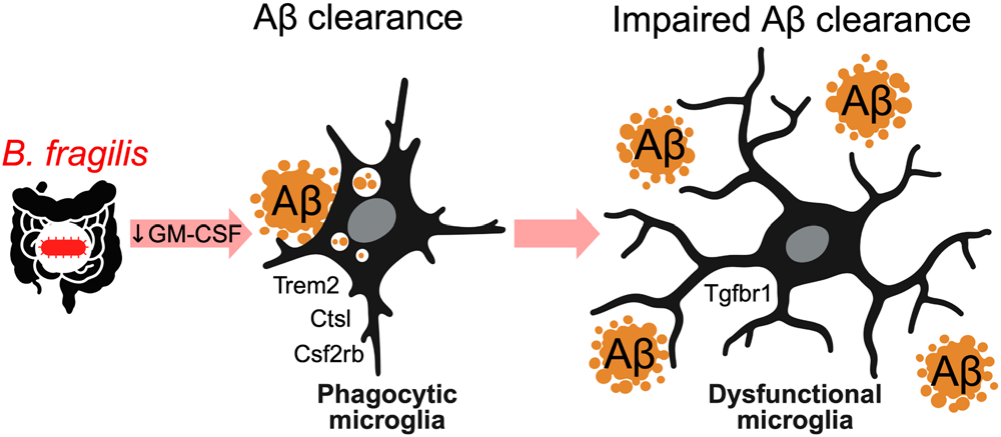
Bacteroides impair microglial-mediated amyloid clearance. Administering *B. fragilis* to mice reduces splenic GM-CSF, reduces expression of microglial genes involved in the response to Aβ, and decreases microglial phagocytic function resulting in impaired Aβ clearance and increased plaques. Created with BioRender.com.

In conclusion, we report that manipulating the microbiota with individual bacteria or a single antibiotic can modulate Aβ pathology in animal models of AD. Our data suggest that gut *B. fragilis* inhibits Aβ clearance by microglia, thus promoting Aβ accumulation in the brain (Fig. 5). The concept that a member of the gut microbiota drives plaque formation in AD by suppressing immunity rather than causing overt inflammation provides a distinct pathway in which a chronic colonization suppresses immune-mediated amyloid clearance. Understanding the mechanisms by which the gut microbiota predisposes to AD will aid the development of microbiota-targeted therapy that may counteract the detrimental role of *B. fragilis* in AD.

## Methods

### Mice

All animal experimental procedures were performed in accordance with the approved Animal Care and Use Protocols. The Institutional Animal Care and Use Committee (IACUC) at Harvard Medical School and Brigham and Women’s Hospital has approved of all procedures involving animals (protocol number N2016000213).

For our experiments we used three transgenic murine models of amyloid pathology that spontaneously develop AD-like disease. For the experiment where mice were administered *B. fragilis*, we used female and male heterozygous APP/PS1-21 Jucker mice on a C57BL/6J background (Supplementary Table 1). APP/PS1 mice carry human APP (coding for the Amyloid precursor protein) and PSEN1 (coding for Presenilin 1 which cleaves APP into aggregation-prone amyloid peptides) transgenes under the Thy1 promoter. These transgenes have mutations associated with familial AD in humans, including the Swedish mutation (K670N/M671L) in APP/PS1 and the L166P mutation in PSEN1, leading to the formation of amyloid plaques starting at 2 months of age. Transgene negative C57BL/6J littermates were used as WT controls. For the experiments where aged mice were administered *B. fragilis*, then injected with Aβ into the brain, we used WT female mice and WT and APPswe/PS1-dE9 male mice (Supplementary Table 2). The APPswe/PS1-dE9 model carry the human APP gene with the Swedish mutation and the human PSEN1 gene lacking exon 9, under the Thy1 promoter. For the experiment where mice were treated with MTZ, we used female heterozygous 5xFAD mice on a C57BL/6 × SJL background (Supplementary Table 3). 5xFAD mice also carry human APP and PSEN1 transgenes under the Thy1 promoter. However, in addition to the Swedish mutation (K670N/M671L) in APP, these mice also have the Florida (I716V) and London (V717I) mutations in APP, and the M146L and L286V mutations in PSEN1, which lead to plaque development in the hippocampus and cortex starting at 1.5 months of age^54^. Transgene negative C57BL/6 × SJL littermates were used as WT controls. Transgenic mice were housed separately from WT mice post weaning to avoid microbiota transfer resulting from coprophagia. The mice were housed in sterilized micro-isolator cages in a biosafety level 2 (BSL2) facility and fed ad libitum on standard chow. The mice were kept on a 12-h light/dark cycle, temperatures were maintained between 19–22 °C and humidity was maintained between 40–60%.

### Oral administration of bacteria

*Bacteroides fragilis* (Type strain, DSM 2151, ATCC 25285, acquired from DSMZ, Braunschweig, Germany), *Faecalibaculum rodentium* (NYU-BL-K8^55^, *Ileibacterium valens* (strain NYU-BL-A3, DSM 103668, ATCC TSD-63) and *Dubosiella newyorkensis* (type strain, NYU-BL-A4, DSM 103457, ATCC TSD-64) were cultured on brucella agar plates (Anaerobe Systems, Morgan Hill, CA, USA) at 37 °C under anaerobic conditions. The inoculum was prepared just before the administration to mice by suspending the bacteria in PRAS Dilution Blanks (Anaerobe Systems) to obtain an optical density of 0.7–0.9 at 600 nm. Our study included three types of experiments that involved administering bacteria to mice. Experiment 1: Two cohorts of female and one cohort of male WT and APP/PS1 mice received oral gavages 1–2 times weekly of *B. fragilis* for 10 weeks starting at 2.6 months of age (Supplementary Table 1 and Supplementary Fig. 2). Experiment 2: Two cohorts of male WT mice (Cohorts 4–5) received a weekly oral gavage of *B. fragilis* for 10 weeks starting either at 8.5 or 12.6 months of age, and one cohort of female WT mice (cohort 6) was received a weekly oral gavage of *B. fragilis* starting at 12.0 months (Supplementary Table 2). Another cohort (cohort 9) of female and male WT mice were treated with a combination of three strains of *Erysipelotrichaceae* ×3 (E3) consisting of *F. rodentium*, *I. valens* and *D. newyorkensis* for 8 weeks, starting at 13.5 months of age (Supplementary Fig. 5A). The mice were treated with 200 µl live bacteria suspension that was administered weekly using reusable stainless-steel feeding needles (20 gauge; 1.5” length; 2.25 mm ball diameter). Mice receiving bacteria were housed in separate cages from PRAS Dilution Blanks (Anaerobe Systems) treated controls.

### Treatment with metronidazole

5xFAD and WT mice (cohort 10) received either normal drinking water or drinking water supplemented with MTZ starting at 9 ±0.8 months of age. MTZ was administered in the drinking water, following the dosing strategy of Minter et al.^30^ with 7 days at a higher dose followed by a lower dose. However, because of concerns of toxicity from metronidazole^56^, we used even lower doses. The first 7 days, the mice received 0.2 mg/mL MTZ in their drinking water followed by a dose of 0.04 mg/mL for an additional 9 weeks. Assuming that a mouse drinks 0.15 L per day, the daily dose was 30 mg/kg the first 7 days and then 6 mg/kg. No weight loss was observed that would indicate reduced intake of water in mice treated with MTZ compared to mice drinking regular water. Fecal pellets were collected from the mice before and 7, 14, 28 and 48 days after the treatment was initiated to analyze changes in the gut bacteria composition in response to MTZ. At the days of euthanasia, samples were also collected from the ileum and cecum.

### Intrahippocampal injections

Mice were anesthetized with either an intraperitoneal injection of ketamine (100 mg/kg) and xylazine (10 mg/kg) or inhalation of isoflurane. Two small holes were drilled in the skull of the mouse 2 millimeters posterior and 1.4 millimeters lateral of bregma (one in each hemisphere). 1 µl of the HiLyte^TM^ Fluor 488 labeled human Aβ-42 peptides (Anaspec, Fremont, CA, USA) was injected 2 millimeters into the holes in the skull to reach the hippocampus using a 5 µL Microliter Syringe model 75 RN with a 28 ga needle (Hamilton, Renado, NV, USA). Postoperative pain was managed with buprenorphine-SR (0.6 mg/kg). The mice were sacrificed 16 h after the injection, and HiLyte^TM^ Fluor 488 positive microglia were analyzed using the staining protocol described for microglia cell sorting described below.

### Immunohistofluorescence

After removal, the left hemisphere was fixed in 4% paraformaldehyde (PFA) overnight, followed by an immersion in a 30% sucrose solution until they sunk. The brain was then frozen in OCT (optimal cutting temperature compound, Sakura Finetek Europe HQ, Alphen aan den Rijn, Netherlands) in a dry-ice ethanol bath, and kept at −30 °C. The hemispheres were then sliced coronally with a cryostat with a width of 25 microns. Because of cutting errors, three mice in cohort 2 were excluded from further analysis. After drying, the slices were kept at −30 °C. Just before staining, tissue sections were thawed and fixed with acetone. After several washes and the sections were blocked with 10% normal horse serum, 2% bovine serum albumin (Sigma-Aldrich, Saint Louis, MO, USA), 1% glycerin and 0.1% Triton X-100 (Sigma-Aldrich) in phosphate-buffered saline (PBS). The slides were then incubated overnight with a goat anti-amyloid clone 6E10 (1:300 dilution, BioLegend, San Diego, CA), which recognizes amino acids 3–8 of Aβ and APP. The next day, the slides were washed again and incubated with a Cy5-anti-goat anti-mouse secondary antibody (BioLegend) for 1 h at room temperature. For microglial analysis, slides were prepared as above and incubated overnight with a mouse anti-amyloid clone 6E10 (1:300 dilution, BioLegend, San Diego, CA), goat anti-Iba1 (1:100 dilution, AbCam, Boston, MA), and rat anti-Clec7a (Invivogen, San Diego, CA). The next day, slides were washed and incubated in AF647-alpaca-anti-mouse, Cy2-donkey-anti-goat, and Cy3-rabbit-anti-rat. The sections were mounted using ProLong Gold Antifade Mountant with DAPI (Invitrogen, Waltham, MA, USA). The images were taken with a Leica DMi8 Widefield microscope (Leica, Wetzlar, Germany), with 5× magnification, an exposure of 300 ms and a light intensity of 81% for DAPI and of 100% for the Cy5 channel for Aβ analysis. For microglia analysis, images were acquired with 10× magnification, an exposure of 100 ms. Using ImageJ^57^, the hippocampus and cortex were analyzed separately. The background was subtracted (0.30) and the threshold was calculated automatically using the triangle algorithm. The percent area covered by the plaques within the delimited surface (cortex or hippocampus) and number of plaques per square millimeter was calculated. Plaque associated microglia was counted automatically by using the ImageJ “Find maxima” function, and the ratio of plaque associated microglia was calculated by dividing the number of microglia in the cortex that overlapped with plaques with the number of microglia not overlapping with plaques, and dividing the number of microglia with total cortex area or total plaque area in the cortex.

### Flow cytometry

The mice were euthanized with CO_2_ until respiratory arrest and perfused before cardiac arrest by injecting 20 ml cold Hank’s buffered saline solution (HBSS) buffer into the left ventricle of the heart. After perfusion, the brain and spleen were removed for fluorescence activated cell sorting (FACS). The brain was homogenized, and immune cells were isolated by gradient centrifugation at 800 × *g* over equal parts of 35% and 70% Percoll (GE Healthcare) at room temperature, the cells were collected from the middle layer. The cells were incubated with antibodies binding CD45-FITC, CD11b-PeCy7, Ly6C-PE and FCRLS-APC for 20 min at room temperature (Supplementary Table 4). Cells were washed with FACS buffer (10% fetal bovine serum, 2.5% 4-(2-hydroxyethyl)-1-piperazineethanesulfonic acid (HEPES) and 0.4% ethylenediaminetetraacetic acid (EDTA) in HBSS), filtered through a 35 µm cell strainer and mixed with 5 µl 7-AAD live-dead stain (Becton, Dickinson and Company, BD, Franklin Lakes, NJ, USA). Microglia was sorted defined as CD45^+^ CD11b^+^ Ly6C^−^, and positive FCLS stain further distinguished microglia from peripherally recruited monocytes^58,59^. 1000 microglia were sorted using an BD FACSAria II cell sorter (BD), and a dry pellet was stored at −80 °C until RNA sequencing (see below).

The spleen was mashed through a 70 μm filter and red blood cells were lysed with ammonium-chloride-potassium (ACK) buffer. The cells were resuspended in complete IMDM medium (10% fetal calf serum (FBS), 1% penicillin-streptomycin, 0.1% β-mercaptoethanol, 1% non-essential amino acids) containing phorbol 12-myristate 13-aceate (PMA, 50 ng/ml, Sigma-Aldrich), ionomycin (1 μM, Sigma-Aldrich) and GolgiStop (1 μg/ml, BD) and incubated for 3 h at 37 °C and 5% CO_2_. After a wash in FACS buffer, the cell suspension was blocked for 20 min using anti-CD16/CD32 Fc-block (BD). Dead cells were stained with Aqua Zombie before fixation (in PBS, 1:1000, BioLegend). Next, the cells were incubated for 20 min at 4 °C with antibodies targeting extracellular proteins (Supplementary Table 1). The antibodies were washed off and the cells were fixed for 20 min in Fixation/Permeabilization buffer (eBioscience Foxp3/Transcription Factor Staining Buffer Set, eBioscience, San Diego, CA, USA). The cells were washed with the Permeabilization buffer and then incubated with intracellular antibodies diluted with the same buffer (Supplementary Table 1). After a wash, cells were resuspended in FACS buffer and analyzed with a BD LSRFortessa flow cytometer (BD) and the FlowJo analysis software (BD), the gating strategies are presented in Fig. 3B and Supplementary Fig. 10.

### RNA sequencing

1000 microglia cells (isolated as described above) were lysed in 5 µl TCL buffer. Preparation of cDNA libraries was performed according to the Smart-seq2 protocol^60^ by the Broad Technology Labs and sequenced by the Broad Genomics Platform. Illumina NextSeq500 and the High Output v2 kit was used to generate 2 ×25 bp reads. Transcripts were quantified by the BTL computational pipeline using Cuffquant version 2.2.1^61^. Raw counts were normalized using DESeq2’s (v1.30.1) median of ratios method^62^. All genes with and average read count of less than 100 reads were removed from the analysis in order to focus on genes with higher relevance for microglia biology, approximately 700–6500 genes were included in the differential expression analyses (Cohort 1 + 2: 703 genes, cohort 3: 5560 genes, cohort 6: 6434 genes, cohort 10: 2970 genes, Supplementary Tables 1–3, respectively). Hypothesis testing was performed by using DESeq2’s Wald test on appropriate variable contrasts. Adjusted *p*-values were determined using the Benjamini–Hochberg method. Pathway analysis was conducted using Ingenuity Pathway Analysis (QIAGEN Inc., https://digitalinsights.qiagen.com/IPA).^63^ for genes with a fold change >±0.2 and a *p*-value < 0.05.

### Microbiome sequencing

DNA was extracted using the DNeasy PowerLyzer DNA isolation kit (Qiagen, Hilden, Germany) following the protocol provided by the manufacturer. The V4 region of the 16S rRNA gene was amplified with barcoded-fusion primers and paired-end 151 base-pair reads were sequenced on the Illumina MiSeq instrument as previously described^64^. Quantitative insights for microbial ecology 2 (Qiime2 v. 2021.8) was used for quality filtering and downstream analysis for compositional analysis^65^. Sequences were filtered for quality by trimming reads below a quality score of q20 and discarding reads shorter than 75% percent of the original length. Sequences were denoised using the DADA2 algorithm^66^ and taxonomy was assigned using the SILVA 138 database (Silva-138-99-515-806-nb-classifier.qza)^67^. Significant differences in taxa in the same mouse over time was calculated with paired Wilcoxon tests. Significant differences in taxa between MTZ treated mice and controls were determined by linear discriminant analysis effect size (LEfSe)^68^.

### NanoString

With the piece of cortex we collected from the right hemisphere, RNA was isolated using mirVana Kit (Ambion, Austin, TX, USA). Gene expression was measured with NanoString neuropathology panel using the nCounter MAX/FLEX System. Using the nSOLVER software, the counts were normalized and genes with an average count above 15 were included in the analysis, a total of 581 genes for *B. fragilis* treated mice and 371 genes for metronidazole treated mice. nSOLVER was also used to generate *p*-values. Pathway analysis was conducted using Ingenuity Pathway Analysis for genes with a fold change >±0.2 and a *p*-value < 0.05.

### Statistics

For statistics related to microglia expression analysis, see section named *RNA sequencing*. For statistics related to expression analysis of cortical tissue, see section named *NanoString*. For statistics related to gut microbial composition, see section named *Microbiome sequencing*.

*P*-values for group comparisons were calculated with two-tailed t-test (amyloid plaque levels, passed the Shapiro–Wilk test for normality), two-tailed Mann–Whitney U test (Aβ uptake by microglia, did not pass the Shapiro–Wilk test for normality) or one-way ANOVA (cytokine production by T cells, passed the Shapiro–Wilk test for normality) using GraphPad Prism 9 for MacOS (v.9.3.1). *P*-values for correlations were calculated with Spearman correlation using GraphPad Prism 9.

In the GM-CSF plot in Fig. 4F, one outlier was removed from the WT-MTZ and 5xFAD-H_2_O groups, and two in the 5xFAD-MTZ group, by two iterations of Grubbs’ test (alpha = 0.05). In Fig. 3C, one outlier was removed from the PBS group using the Grubbs’ method, alpha = 0.05.

In all violin plots, the dots represent distinct samples and specifies the number of observations in each group. The lines represent median and interquartile range. All central tendencies reported in the main text are means, unless stated otherwise.

Plots were made in R Studio (v.2022.02.1 Build 461) using the ggplot2 package (boxplots), base R (scatterplots) and the DESeq2 package (PCA plots) in R v.4.1.2, or GraphPad Prism (violin plots and heatmaps) and figures were constructed in Adobe Illustrator.

### Reporting summary

Further information on research design is available in the Nature Portfolio Reporting Summary linked to this article.

### Supplementary information

Supplementary Material

Reporting Summary

### Source data

Source Data

## Data Availability

The microglia RNA-sequencing data and microbiome 16 S rRNA sequencing data generated in this study have been deposited in the National Center for Biotechnology Information (NCBI) Short Read Archive (SRA) under accession code PRJNA876228 for our *Bacteroides* investigations and PRJNA876231 for our metronidazole investigations. Source data are provided with this paper. The processed data generated from histology, flow cytometry and NanoString are provided in the Source Data file. The Source Data file also includes lists of significant genes from RNA sequencing experiments.
