# Supplementary material for: Bacteroidota inhibit microglia clearance of amyloid-beta and promote plaque deposition in Alzheimer’s disease mouse models
Source: Nat Commun. 2024 May 8;15:3872. doi: 10.1038/s41467-024-47683-w (PMC11078963; doi:10.1038/s41467-024-47683-w)
Supplement: Supplementary file 1 — Supplementary Material [file 41467_2024_47683_MOESM1_ESM.pdf]

Supplementary Material

Bacteroidota inhibit microglia clearance of amyloid-beta and promote plaque deposition in Alzheimer's disease mouse models

Caroline Wasén[1,2,3], Leah C. Beauchamp[1], Julia Vincentini[1], Shuqi Li[1], Danielle S. LeServe[1], Christian Gauthier[1], Juliana R. Lopes[1], Thais G. Moreira[1], Millicent Ekwudo[1], Zhuoran Yin[1,4], Patrick Da Silva, Rajesh K. Krishnan[1], Oleg Butovsky[1], Laura M. Cox[1*], Howard L. Weiner[1*]

[1] Ann Romney Center for Neurologic Diseases, Brigham & Women's Hospital, Harvard Medical School, Boston, MA, USA

[2]Department of Biology and Biological Engineering, Chalmers University of Technology, Gothenburg, Sweden

[3]Department of Rheumatology and Inflammation Research, Sahlgrenska Academy, University of Gothenburg, Gothenburg, Sweden

[4]Department of Ophthalmology, Massachusetts Eye and Ear, Harvard Medical School, Boston, MA, USA

[*]Corresponding authors: lcox@bwh.harvard.edu; hweiner@rics.bwh.harvard.edu

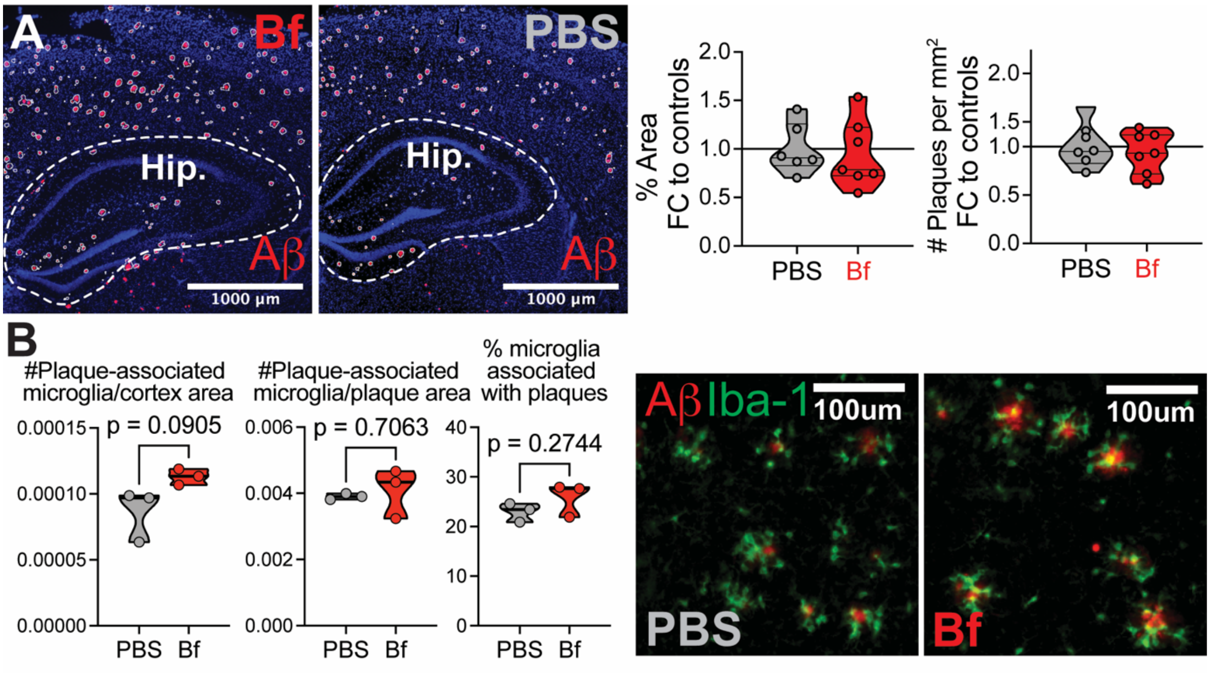

Supplementary figure 1. Amyloid plaque burden in hippocampus of mice treated with *Bacteroides fragilis* (Bf). Amyloid plaque burden and Iba-1$^+$ microglia were assessed in mice treated with Bf by immunohistofluorescence. **A**) Plaque burden in the hippocampus was defined as the percent of the cortex area that were covered with plaques and the number of plaques per square millimeter in the cortex. N=6-7, the data is pooled from two independent experiments. **B**) Number of plaque-associated microglia per total area of the cortex selection and per total area of amyloid plaques in the cortex, and the percentage of microglia in the cortex that were associated with plaques. N=3 mice/group (cohort 2). P-values were calculated with Student's t-test. Violin plots represent min, max, interquartile range and median, the dots represent mice.

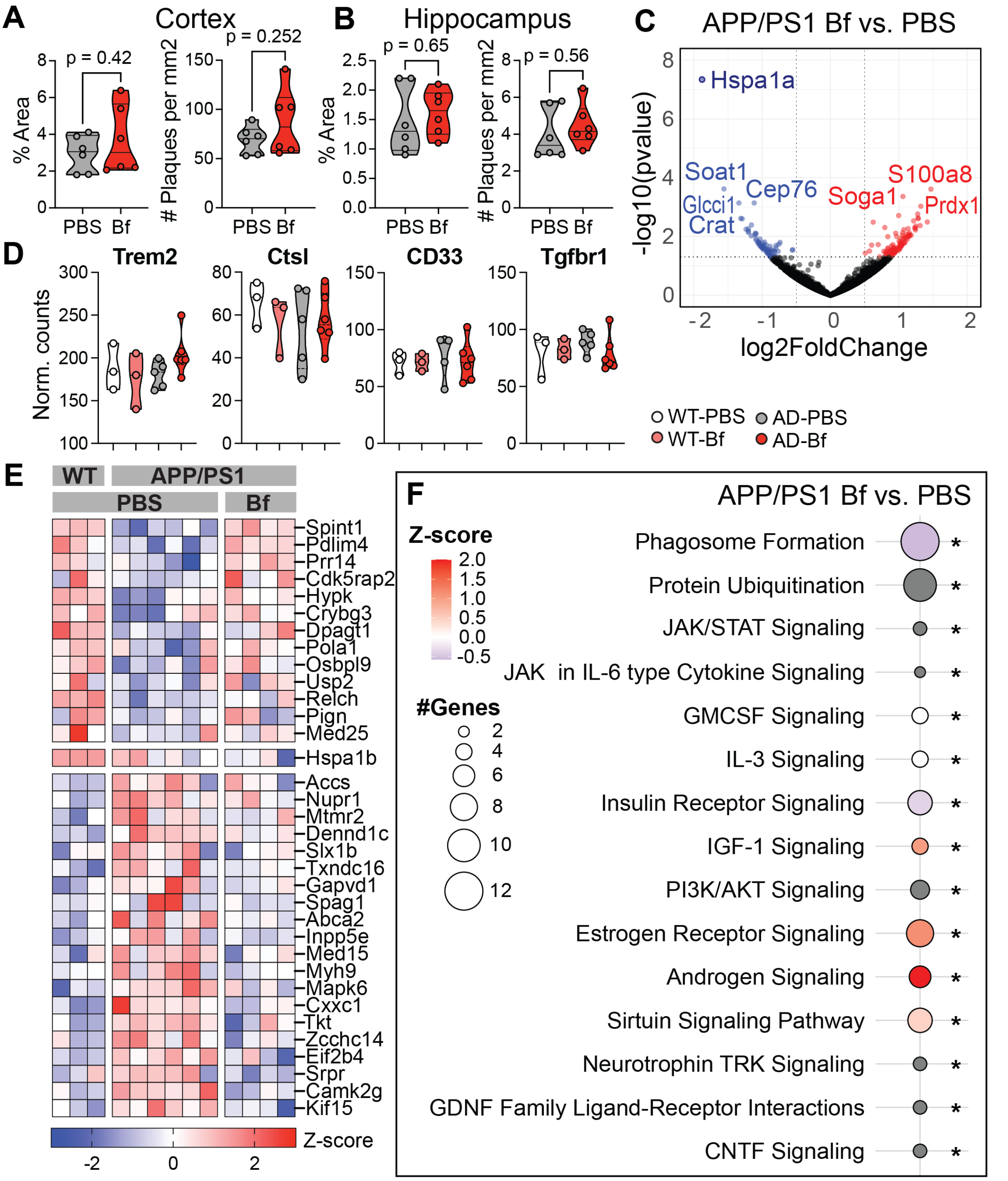

Supplementary figure 2. The effect on *Bacteroides fragilis* (Bf) on amyloid plaques and microglia gene expression in male mice. A) Wildtype (WT) and APP/PS1 male mice were treated with Bf biweekly between 2.9 and 5.7 months of age. **B**) Percent area of parietal cortex and hippocampus that was covered in plaques and number of plaques per mm^2. **C**) Volcano plot of microglia gene expression. **D**) Normalized counts of genes related to homeostatic and neurodegenerative microglia signatures. **E**) Genes with a $p<0.05$ in both APP/PS1 vs. WT (PBS treated) and Bf vs. PBS (APP/PS1). **F**) Pathways altered by Bf in APP/PS1 mice identified by Ingenuity Pathway Analysis (IPA). Genes with a FC >±0.2 and an unadjusted p-value <0.05 were included in the analysis. Circles are shaded according to the z-score indicating activation (red) or inhibition (blue) or no presumed direction (grey). Violin plots represent min, max, interquartile range and median, the dots represent mice.

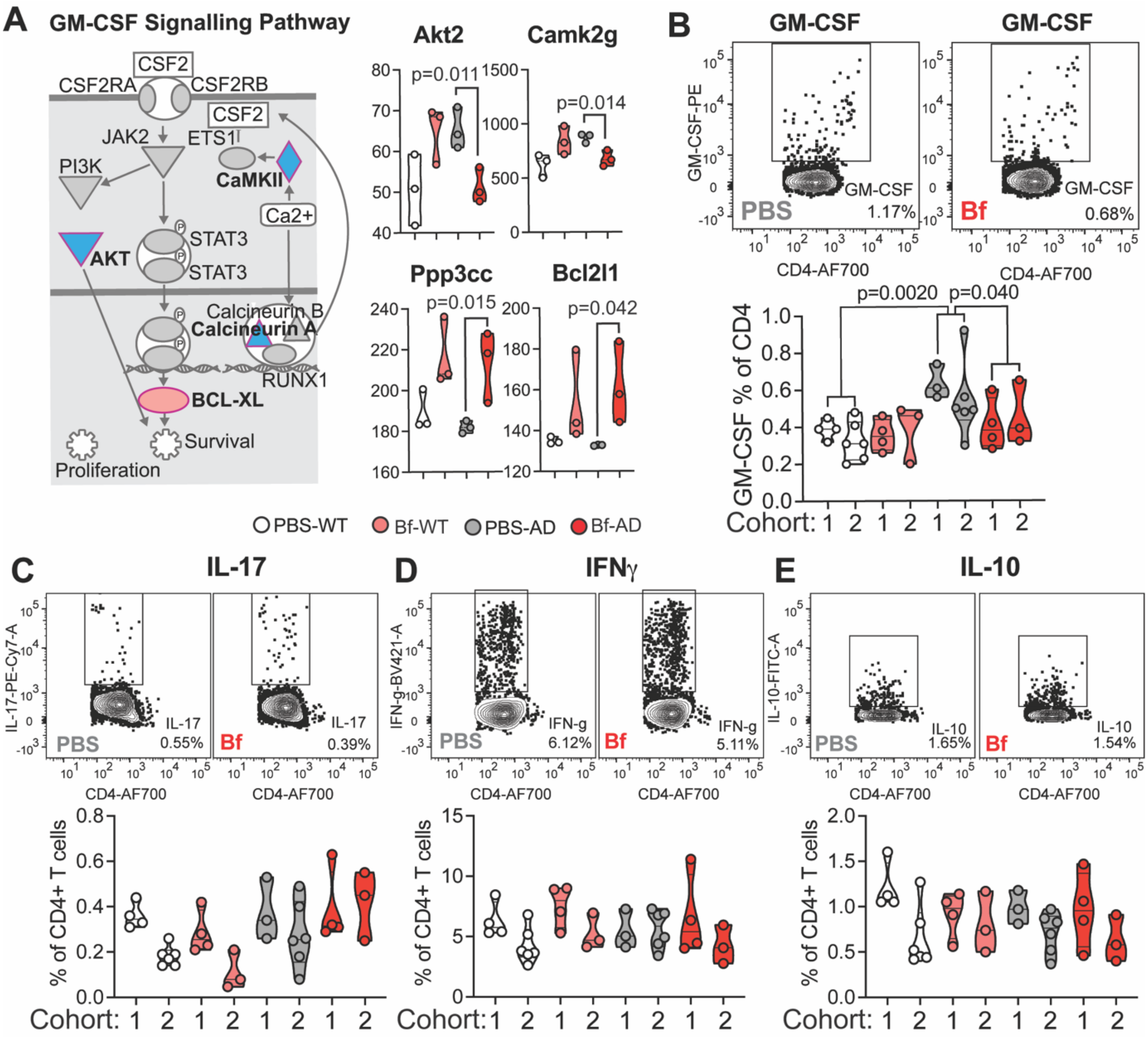

Supplementary figure 3. *Bacteroides fragilis* suppress GM-CSF signaling in peripheral T cells and cortical tissue. A) Schematic figure of the GM-CSF signaling pathway (adaption of IPA-generated graph) and graphs showing the differentially expressed genes in the pathways, the p-values were generated with NSolver Advanced Analysis. n=3 mice/group. **B-E**) GM-CSF (**B**), IL-17 (**C**), IFN-g (**D**) and IL-10 (**E**) production by splenic CD4^{+} T cells stimulated for 4h with PMA/ionomycin. The data is pooled from two independent experiments. P-values are calculated with one-way ANOVA, n=6-7 mice/group. Violin plots represent min, max, interquartile range and median, the dots represent mice.

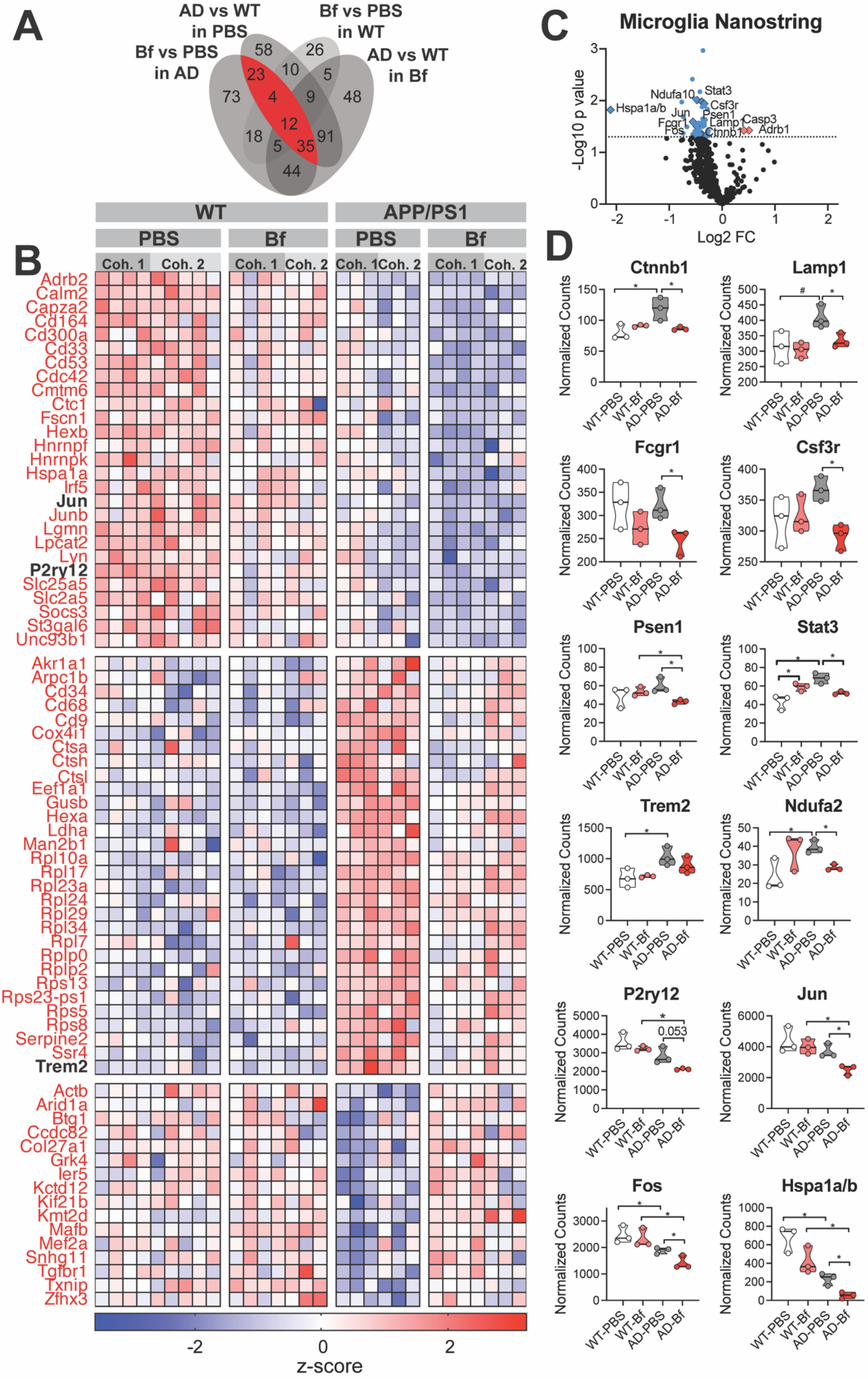
A
AD vs WT in PBS
Bf vs PBS in WT
Bf vs PBS in AD
AD vs WT in Bf
58
26
23
10
5
73
4
9
48
12
18
91
5
35
44
B
WT
APP/PS1
PBS
Bf
PBS
Bf
Coh. 1
Coh. 2
Adrb2
Calm2
Capza2
Cd164
Cd300a
Cd33
Cd53
Cdc42
Cmtm6
Ctc1
Fscn1
Hexb
Hnrnpf
Hnrnpk
Hspa1a
Irf5
Jun
Junb
Lgmn
Lpcat2
Lyn
P2ry12
Slc25a5
Slc2a5
Socs3
St3gal6
Unc93b1
Akr1a1
Arpc1b
Cd34
Cd68
Cd9
Cox4i1
Ctsa
Ctsh
Ctsl
Eef1a1
Gusb
Hexa
Ldha
Man2b1
Rpl10a
Rpl17
Rpl23a
Rpl24
Rpl29
Rpl34
Rpl7
Rplp0
Rplp2
Rps13
Rps23-ps1
Rps5
Rps8
Serpine2
Ssr4
Trem2
Actb
Arid1a
Btg1
Ccdc82
Col27a1
Grk4
Ier5
Kctd12
Kif21b
Kmt2d
Mafb
Mef2a
Snhg11
Tgfbr1
Txnip
Zfhx3
-2
0
2
z-score
C
Microglia Nanostring
-Log10 p value
Log2 FC
Hspa1a/b
Ndufa10
Stat3
Csf3r
Jun
Psen1
Fcgr1
Lamp1
Casp3
Fos
Ctnnb1
Adrb1
D
Ctnnb1
Lamp1
Fcgr1
Csf3r
Psen1
Stat3
Trem2
Ndufa2
P2ry12
Jun
Fos
Hspa1a/b
Normalized Counts
WT-PBS
WT-Bf
AD-PBS
AD-Bf
0.053

Supplementary figure 4. Microglia gene expression in mice treated with *B. fragilis*. Microglia were isolated from APP/PS1 (Alzheimer's disease, AD) and wild type (WT) female mice treated with *B. fragilis* (Bf) between 2 and 5 months of age and analyzed by RNA sequencing and NanoString Microglia Panel. The Venn-diagram **(A)** show the number of differentially expressed genes (DEGs). The heatmap **(B)** show DEGs that were different in both Bf vs. PBS in APP/PS1 and WT vs. APP/PS1 treated with PBS. Transcripts with an average count of 100 (scaled by size factors) across all samples were included in the analyses. **C**) Volcano plot of microglia analyzed by NanoString from cohort 2 APP/PS1 females treated with B. fragilis vs. PBS (control). **D**) Violin plots of microglia gene expression measured by NanoString, the p-values were generated with NSolver Advanced Analysis. n=3 mice/group. Violin plots represent min, max, interquartile range and median, the dots represent mice.

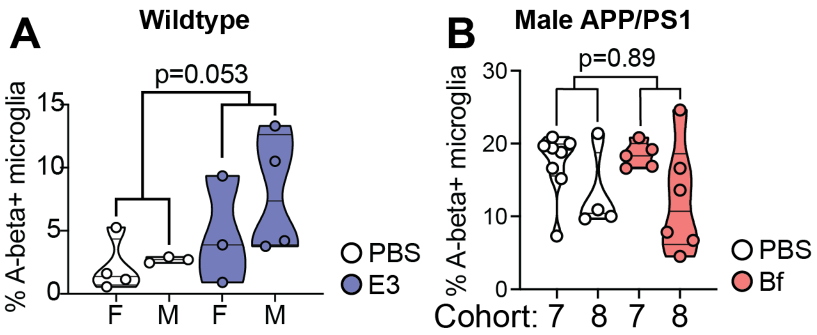

Supplementary figure 5. Amyloid-β uptake after treatment with *Erysipelotrichaceae* and after treatment with *Bacteroides fragilis* (Bf) in APP/PS1 mice. APP/PS1 mice were treated with Bf and wildtype (WT) mice were treated with *Erysipelotrichaceae x3* (E3) by weekly gavages before amyloid-β injection into the hippocampus and sacrifice. **A-B)** Amyloid-**β** uptake after E3 administration in pooled data of females and males (**A**, cohort 9) and after Bf administration in cohort 7 and cohort 8 (**B**). P-values were calculated using the Mann-Whitney U test. N=7-12 mice/group. Violin plots represent min, max, interquartile range and median, the dots represent mice.

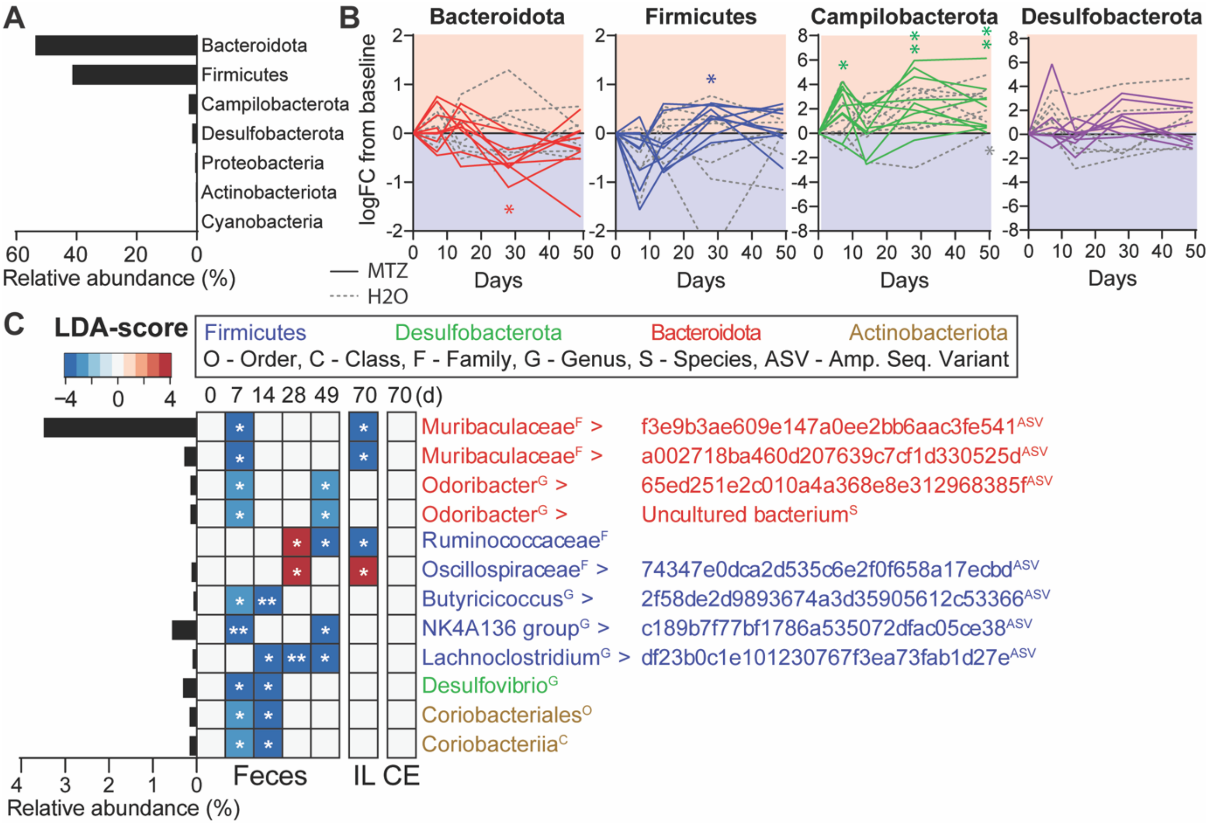

Supplementary figure 6. The effect of metronidazole on the relative abundance of bacteria in the gut. Female and 5xFAD and wild type (WT) mice were treated with metronidazole (MTZ) in their drinking water between 9 and 12 months of age, controls were drinking regular water (H2O). Feces were collected before and after 7, 14, 28 and 49 days of MTZ treatment. Ileum (IL) and cecum (CE) samples were collected when the mice were sacrificed, 70 days after MTZ treatment started. **A**) The average relative abundance of bacteria at phylum taxonomic level in feces. **B**) The logarithm of the fold change (logFC) relative abundance of the four most abundant phyla compared to the abundance before initiating MTZ treatment. P-values are calculated with Wilcoxon paired analysis. **C**) The heatmap show differences in the relative abundance of bacteria in feces, IL and CE of 5xFAD mice at different timepoints after initiation of MTZ treatment, bacteria that were different from controls at least at two time points were included in the graph. A lower Linear Discriminatory Analysis (LDA) score indicates a lower relative abundance. *p<0.05, **p<0.01.

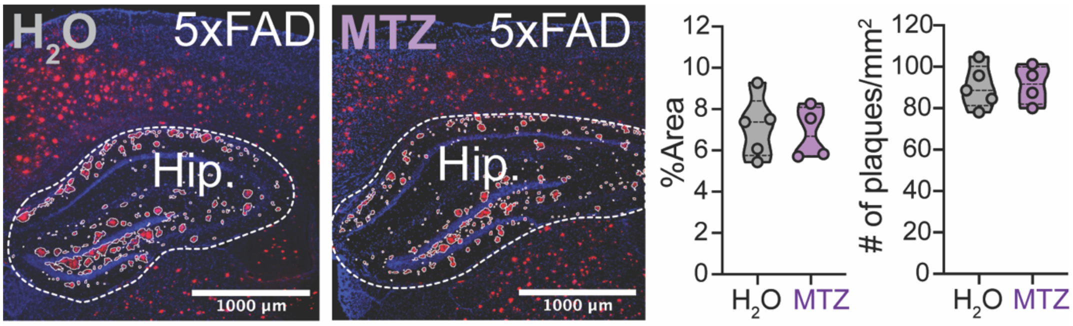

Supplementary figure 7. Amyloid plaque burden in hippocampus of mice treated with metronidazole (MTZ). Amyloid plaque burden was assessed in mice treated with MTZ by immunohistofluorescence of the hippocampus. Plaque burden is defined as the percent of the cortex area that were covered with plaques and the number of plaques per square millimeter in the cortex. N=4-5 mice/group. Violin plots represent min, max, interquartile range and median, the dots represent mice.

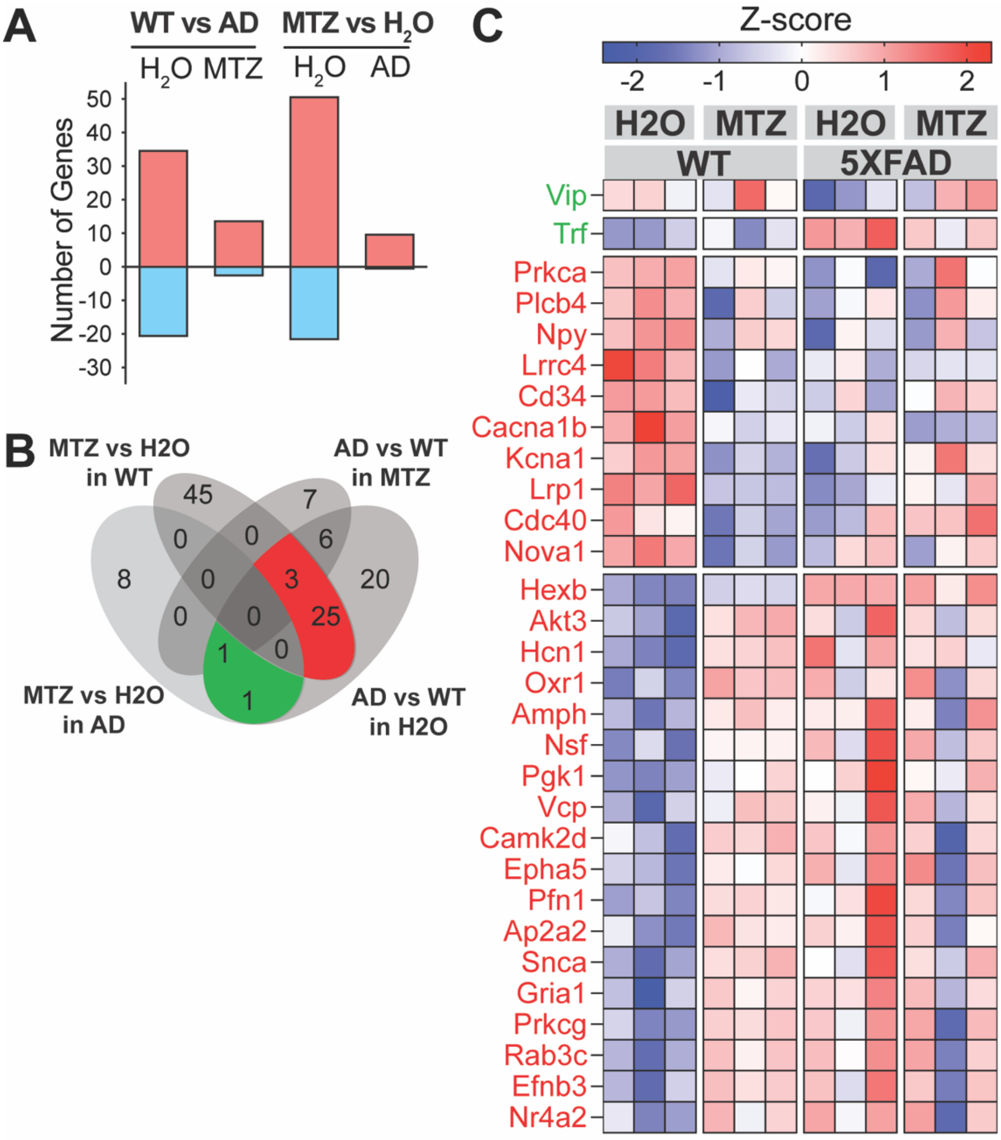

Supplementary figure 8. Metronidazole (MTZ) affects cortical gene expression. Cortical tissue from female 5xFAD and wild type (WT) mice treated with MTZ between 9 and 12 months of age was analyzed for transcriptional changes with the NanoString Neuropathology panel. **A)** Number of up or down regulated genes in cortex. **B**) Venn diagram showing the overlap in differentially expressed genes with a fold change (FC) greater than 0.2 and a p-value below 0.05. **C**) Heatmap showing the overlapping genes highlighted in figure B.

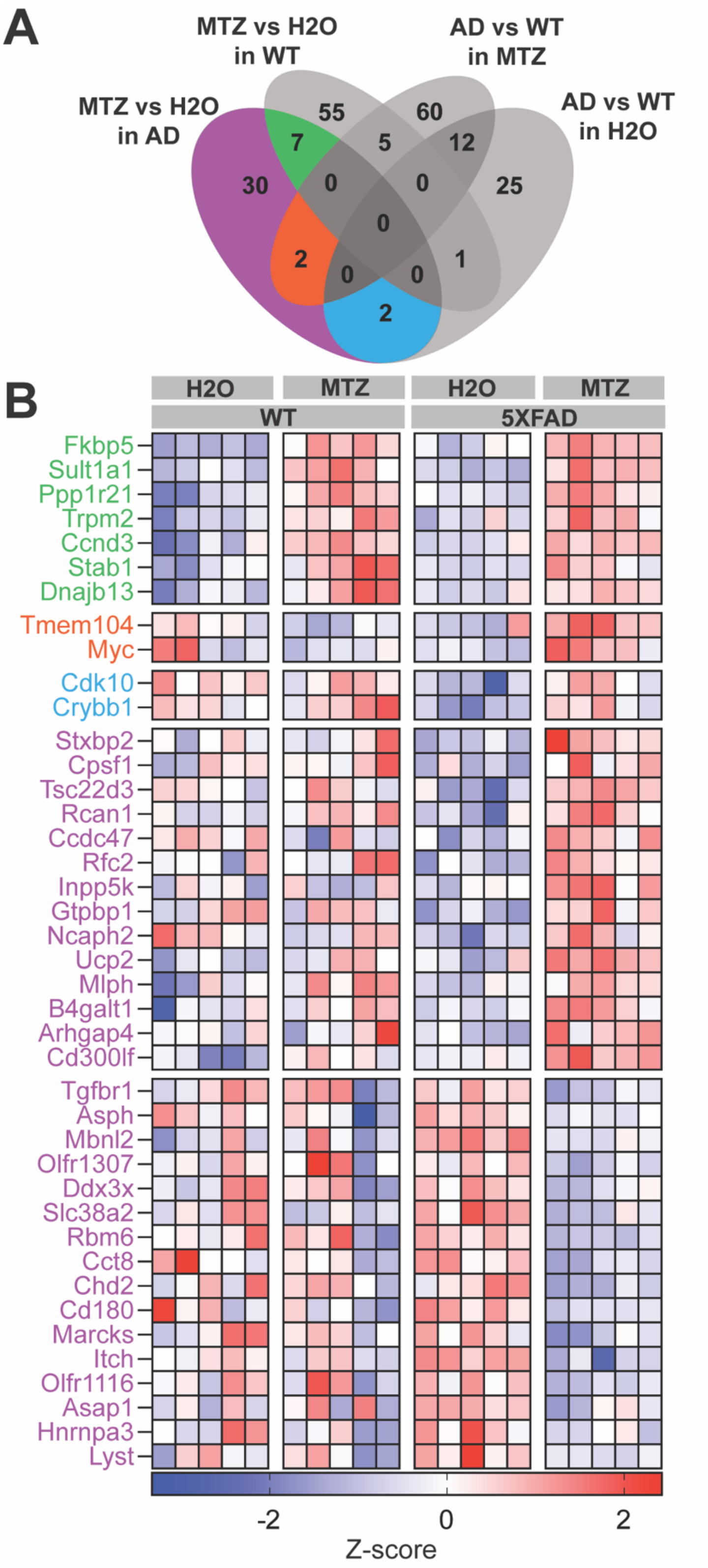

Supplementary figure 9. Microglia gene expression in mice treated with metronidazole. Microglia were isolated from 5xFAD and wild type (WT) mice treated with metronidazole (MTZ) at 9-12 months of age and analyzed by RNA sequencing. The Venn-diagram **(A)** show the number of DEGs. The heatmap **(B)** show DEGs indicated by color in the Venn-diagram **(A)**. Transcripts with an average count of 100 (scaled by size factors) across all samples were included in the analyses.

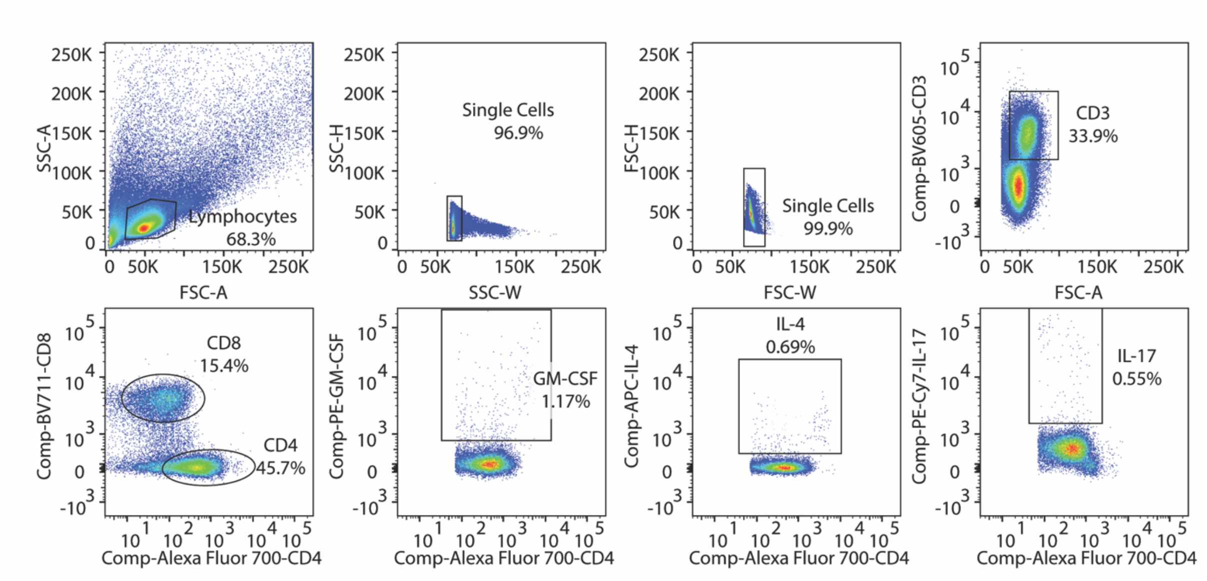

Supplementary figure 10. Gating strategy. Gating strategy for GM-CSF production by CD4 T cells.

Supplementary table 1. Experimental cohorts to investigate plaque burden and microglia transcriptomics

Cohort	Sex	Genotype	Treatment	Age start (m) mean (range)	Age sacrifice (m) mean (range)
1	Female	APP/PS1-21	PBS (n=3)	2.63 (2-3.07)	4.95 (4.33-5.41)
	Female	APP/PS1-21	Bf (n=4)	2.45 (2-3.07)	5.02 (4.52-5.67)
	Female	Wildtype	PBS (n=4)	2.63 (2-3.07)	4.84 (4.2-5.25)
	Female	Wildtype	Bf (n=4)	2.68 (2-3.07)	5.11 (4.49-5.44)
2	Female	APP/PS1-21	PBS (n=3)	2.73 (2.46-3.05)	5.49 (5.21-5.8)
	Female	APP/PS1-21	Bf (n=3)	2.63 (2.52-2.89)	5.39 (5.28-5.64)
	Female	Wildtype	PBS (n=3)	2.69 (2.46-3.05)	5.45 (5.21-5.8)
	Female	Wildtype	Bf (n=3)	2.73 (2.46-3.05)	5.48 (5.21-5.8)
3	Male	APP/PS1-21	PBS (n=6)	2.79 (2.52-3.05)	5.55 (5.28-5.8)
	Male	APP/PS1-21	Bf (n=7)	2.79 (2.46-3.05)	5.55 (5.21-5.64)
	Male	Wildtype	PBS (n=3)	3.04 (2.46-3.61)	5.79 (5.21-6.36)
	Male	Wildtype	Bf (n=4)	3.1 (2.56-3.64)	5.85 (5.31-3.69)

PBS = Phosphate buffered saline, vehicle control. Bf = Bacteroides fragilis

Supplementary table 2. Experimental cohorts to investigate Aβ uptake by microglia

Cohort	Sex	Genotype	Treatment	Age start (m) mean (range)	Age sacrifice (m) mean (range)
4	Male	Wildtype	PBS (n=3)	12.6 (12.4-12.7)	15.0 (14.7-15.2)
	Male	Wildtype	Bf (n=4)	12.5 (12.2-12.7)	14.9 (14.7-15.2)
5	Male	Wildtype	PBS (n=7)	8.6 (8.5-8.6)	11.56 (11.51-11.57)
	Male	Wildtype	Bf (n=6)	8.4 (8.20-8.6)	11.35 (11.18-11.54)
6	Female	Wildtype	PBS (n=5)	11.8 (11.7-11.9)	14.2 (14.1-14.3)
	Female	Wildtype	Bf (n=7)	11.8 (11.7-11.9)	14.2 (14.1-14.3)
7	Male	APPS/PS1-dE9	PBS (n=5)	12.6 (12.4-12.7)	14.8 (14.5-14.9)
	Male	APPS/PS1-dE9	Bf (n=5)	12.5 (12.2-12.7)	14.7 (14.5-14.9)
8	Male	APPS/PS1-dE9	PBS (n=3)	8.3 (7.5-8.6)	11.0 (10.5-11.3)
	Male	APPS/PS1-dE9	Bf (n=4)	8.4 (8.1-8.5)	11.2 (10.9-11.4)
9	Female	Wildtype	PBS (n=5)	14.0 (13.2-14.9)	16.0 (15.2-16.9)
	Female	Wildtype	E3 (n=5)	14.0 (13.2-14.9)	16.0 (15.2-16.9)
	Male	Wildtype	PBS (n=3)	13.5 (13.2-14.0)	15.5 (15.2-16.0)
	Male	Wildtype	E3 (n=4)	13.8 (13.2-14.9)	15.8 (15.2-16.9)

PBS = Phosphate buffered saline, vehicle control. Bf = Bacteroides fragilis. E3 = 3 strains of Erysipelotrichaceae.

Supplementary table 3. Experimental cohort to investigate metronidazole

Cohort	Sex	Genotype	Treatment	Age start (m) mean (range)	Age sacrifice (m) mean (range)
10	Female	5xFAD	H2O (n=8)	9.23 (6.67-11.13)	11.47 (8.95-13.34)
	Female	5xFAD	MTZ (n=9)	9.44 (6.67-11.13)	11.68 (8.95-13.34)
	Female	Wildtype	H2O (n=7)	9.07 (8.2-9.67)	11.31 (10.46-11.9)
	Female	Wildtype	MTZ (n=8)	9.34 (6.67-11.47)	11.53 (8.95-13.67)

MTZ = Metronidazole

Supplementary table 4. Antibodies

Target	Fluorophore	Clone	Company	Catalog #	Dilution	Location	Tissue
CD3	BV605	17A2	Biolegend	100237	1:400*	Extracellular	Spleen
CD4	BV785	RM4-5	Biolegend	100506	1:100*	Extracellular	Spleen
CD8a	BV711	53-6.7	BD	563046	1:400**	Extracellular	Spleen
IFN-γ	BV421	XMG1.2	Biolegend	505830	1:400*	Intracellular	Spleen
IL-17A	PE-Cy7	eBio17B7	ThermoFisher	25-7177-82	1:400*	Intracellular	Spleen
IL-10	FITC	JES5-16E3	Biolegend	505006	1:100*	Intracellular	Spleen
GM-CSF	PE	MP1-22E9	ThermoFisher	12-7331-82	1:100*	Intracellular	Spleen
CD45	FITC	30-F11	eBioscience	11-0451-85	1:400*	Extracellular	Brain
CD11b	PE-Cy7	M1/70	eBioscience	25-0112-82	1:400*	Extracellular	Brain
Ly-6C	PE	HK1.4	eBioscience	12-5932-82	1:400*	Extracellular	Brain
FCRLS	APC	4G11	Butovsky Lab		1:1000*	Extracellular	Brain
TCR-β	PerCp	H57-597	Biolegend	109228	1:800*	Extracellular	Brain
B220	PerCp	RA3-6B2	Invitrogen	45-0452-82	1:800*	Extracellular	Brain
NK1.1	PerCp	PK136	Biolegend	108726	1:800*	Extracellular	Brain
CD317	PerCp	927	Biolegend	127022	1:800*	Extracellular	Brain
Ly-6G	PerCp	1A8	Biolegend	127654	1:800*	Extracellular	Brain

*Dilution determined by titration.
