# Supplementary material for: Bacteroidota inhibit microglia clearance of amyloid-beta and promote plaque deposition in Alzheimer’s disease mouse models
Source: Nat Commun. 2024 May 8;15:3872. doi: 10.1038/s41467-024-47683-w (PMC11078963; doi:10.1038/s41467-024-47683-w)
Supplement: Supplementary file 2 — Reporting Summary [file 41467_2024_47683_MOESM2_ESM.pdf]

nature portfolio

Corresponding author(s): Laura Cox
Howard Weiner

Last updated by author(s): 27/02/23

Reporting Summary

Nature Portfolio wishes to improve the reproducibility of the work that we publish. This form provides structure for consistency and transparency in reporting. For further information on Nature Portfolio policies, see our Editorial Policies and the Editorial Policy Checklist.

Statistics

For all statistical analyses, confirm that the following items are present in the figure legend, table legend, main text, or Methods section.

n/a	Confirmed	
☐	☒	The exact sample size (*n*) for each experimental group/condition, given as a discrete number and unit of measurement
☐	☒	A statement on whether measurements were taken from distinct samples or whether the same sample was measured repeatedly
☐	☒	The statistical test(s) used AND whether they are one- or two-sided<br>*Only common tests should be described solely by name; describe more complex techniques in the Methods section.*
☒	☐	A description of all covariates tested
☐	☒	A description of any assumptions or corrections, such as tests of normality and adjustment for multiple comparisons
☐	☒	A full description of the statistical parameters including central tendency (e.g. means) or other basic estimates (e.g. regression coefficient) AND variation (e.g. standard deviation) or associated estimates of uncertainty (e.g. confidence intervals)
☐	☒	For null hypothesis testing, the test statistic (e.g. *F*, *t*, *r*) with confidence intervals, effect sizes, degrees of freedom and *P* value noted<br>*Give P values as exact values whenever suitable.*
☒	☐	For Bayesian analysis, information on the choice of priors and Markov chain Monte Carlo settings
☒	☐	For hierarchical and complex designs, identification of the appropriate level for tests and full reporting of outcomes
☐	☒	Estimates of effect sizes (e.g. Cohen's *d*, Pearson's *r*), indicating how they were calculated

Our web collection on statistics for biologists contains articles on many of the points above.

Software and code

Policy information about availability of computer code

Data collection

No software was used.

Data analysis

RNA-seq: Transcripts were quantified by the BTL computational pipeline using Cuffquant version 2.2.1 55. Raw counts were normalized using DESeq2's (v1.30.1) median of ratios method. All genes with and average read count of less than 100 reads were removed from the analysis. Hypothesis testing was performed by using DESeq2's Wald test on appropriate variable contrasts. Adjusted p-values were determined using the Benjamini-Hochberg method. Pathway analysis was conducted using Ingenuity Pathway Analysis for genes with a fold change >±0.2 and a p-value <0.05.

16S sequencing: Quantitative insights for microbial ecology 2 (Qiime2 v. 2021.8) was used for quality filtering and downstream analysis for compositional analysis. Sequences were filtered for quality by trimming reads below a quality score of q20 and discarding reads shorter than 75% percent of the original length. Sequences were denoised using the DADA2 algorithm and taxonomy was assigned using the SILVA 138 database (Silva-138-99-515-806-nb-classifier.qza). Significant differences in taxa in the same mouse over time was calculated with paired Wilcoxon tests. Significant differences in taxa between MTZ treated mice and controls were determined by linear discriminant analysis effect size (LEfSe).

NonoString: gene expression was measured with NanoString neuropathology panel using the nCounter MAX/FLEX System. Using the nSOLVER software, the counts were normalized and genes with an average count above 15 were included in the analysis,. nSOLVER was also used to generate p-values. Pathway analysis was conducted using Ingenuity Pathway Analysis for genes with a fold change >±0.2 and a p-value <0.05.

Other statistics: P-values for group comparisons were calculated with two-tailed t-test (amyloid plaque levels, passed the Shapiro-Wilk test for

normality), two-tailed Mann-Whitney U test (A-beta uptake by microglia, did not pass the Shapiro-Wilk test for normality) or one-way ANOVA (cytokine production by T cells, passed the Shapiro-Wilk test for normality) using GraphPad Prism 9 for MacOS (v.9.3.1). P-values for correlations were calculated with Spearman correlation using GraphPad Prism 9.

For manuscripts utilizing custom algorithms or software that are central to the research but not yet described in published literature, software must be made available to editors and reviewers. We strongly encourage code deposition in a community repository (e.g. GitHub). See the Nature Portfolio guidelines for submitting code & software for further information.

Data

Policy information about availability of data

All manuscripts must include a data availability statement. This statement should provide the following information, where applicable:

- Accession codes, unique identifiers, or web links for publicly available datasets
- A description of any restrictions on data availability
- For clinical datasets or third party data, please ensure that the statement adheres to our policy

The microglia RNA-sequencing data and microbiome 16S rRNA sequencing data generated in this study have been deposited in the Short Read Archive (SRA) under project numbers PRJNA876228 for our Bacteroides investigations and PRJNA876231 [https://www.ncbi.nlm.nih.gov/bioproject/?term=PRJNA876231] for our metronidazole investigations. Source data are provided with this paper. The processed data generated from histology, flow cytometry and NanoString are provided in the Source Data file. The Source Data file also includes lists of significant genes from RNA sequencing experiments.

Research involving human participants, their data, or biological material

Policy information about studies with human participants or human data. See also policy information about sex, gender (identity/presentation), and sexual orientation and race, ethnicity and racism.

Reporting on sex and gender	NA.
Reporting on race, ethnicity, or other socially relevant groupings	NA.
Population characteristics	NA.
Recruitment	NA.
Ethics oversight	NA.

Note that full information on the approval of the study protocol must also be provided in the manuscript.

Field-specific reporting

Please select the one below that is the best fit for your research. If you are not sure, read the appropriate sections before making your selection.

- [x] Life sciences
- [ ] Behavioural & social sciences
- [ ] Ecological, evolutionary & environmental sciences

For a reference copy of the document with all sections, see nature.com/documents/nr-reporting-summary-flat.pdf

Life sciences study design

All studies must disclose on these points even when the disclosure is negative.

Sample size	Sample sizes were chosen based on data reported in Cox et al. 2019 in wihch group sizes of 4-5 mice were sufficient to observe statistically significant results with regard to amyloid plaque deposition in APP/PS1 mice treated with B. fragilis.
Data exclusions	In the GM-CSF plot in Figure 4F, one outlier was removed from the WT-MTZ and 5xFAD-H2O groups, and two in the 5xFAD-MTZ group, by two iterations of Grubbs' test (alpha=0.05). In Figure 3C, one outlier was removed from the PBS group using the Grubbs' test, alpha=0.05.
Replication	Effects of B. fragilis on amyloid plaque deposition and microglia transcriptional responses: three cohorts were used - two independent experiments with female WT mice, one with male WT mice and one with male APP/PS1 mice. The female cohorts both had increased plaque burden in response to B. fragilis administration and similar microglia responses based on RNA sequencing, including reduced Trem2 and Ctsl expression and increased expression of Tgfbr1. In male mice, we observed similar trends with regard to phagocytosis pathways as in females, but no difference in plaque burden.<br><br>Effects of B. fragilis on microglia uptake by microglia: four cohorts were used - two independent experiments with male WT mice, one experiment with female animals and one experiment with male APP/PS1 mice. When B.fragilis were given to WT mice that were later injected with amyloid beta, we observed reduced microglia uptake in both male and female cohorts. However, when amyloid beta was injected to APP/PS1 mice who already had advance amyloid pathology, we obseved no difference in microglia phagocytosis.
Randomization	Because transgenic AD models develop amyloid plaques over time, all experimental groups were age-matched. To avoid bias, mice from different litters were distributed evenly over experimental groups. After age of weaning, mice of different genotypes were not co-housed as WT gut microbiome has been shown to affect amyloid pathology in AD models.

Blinding	At the time for organ collection, tissue processing and analysis, the mice were given a new ID number and all investigators involved in harvesting organs and preparing them for analysis were unaware of group allocation. The data generated by RNA sequencing, 16S sequencing, NanoString, FACS and image analysis remained blinded until the time for statistical testing.

Reporting for specific materials, systems and methods

We require information from authors about some types of materials, experimental systems and methods used in many studies. Here, indicate whether each material, system or method listed is relevant to your study. If you are not sure if a list item applies to your research, read the appropriate section before selecting a response.

Materials & experimental systems

n/a	Involved in the study
☐	☒ Antibodies
☒	☐ Eukaryotic cell lines
☒	☐ Palaeontology and archaeology
☐	☒ Animals and other organisms
☒	☐ Clinical data
☒	☐ Dual use research of concern
☒	☐ Plants

Methods

n/a	Involved in the study
☒	☐ ChIP-seq
☐	☒ Flow cytometry
☒	☐ MRI-based neuroimaging

Antibodies

Antibodies used

Target Fluorophore Clone Company Catalog# Dilution Location Tissue
CD3 BV605 17A2 Biolegend 100237 1:400 Extracellular Spleen
CD4 BV785 RM4-5 Biolegend 100506 1:100 Extracellular Spleen
CD8a BV711 53-6.7 BD 563046 1:400 Extracellular Spleen
IFN-γ BV421 XMG1.2 Biolegend 505830 1:400 Intracellular Spleen
IL-17A PE-Cy7 eBio17B7 ThermoFisher 25-7177-82 1:400 Intracellular Spleen
IL-10 FITC JES5-16E3 Biolegend 505006 1:100 Intracellular Spleen
GM-CSF PE MP1-22E9 ThermoFisher 12-7331-82 1:100 Intracellular Spleen
CD45 FITC 30-F11 eBioscience 11-0451-85 1:400 Extracellular Brain
CD11b PE-Cy7 M1/70 eBioscience 25-0112-82 1:400 Extracellular Brain
Ly-6C PE HK1.4 eBioscience 12-5932-82 1:400 Extracellular Brain
FCRLS APC 4G11 Butovsky Lab 1:1000 Extracellular Brain
TCR-beta PerCp H57-597 Biolegend 109228 1:800 Extracellular Brain
B220 PerCp RA3-6B2 Invitrogen 45-0452-82 1:800 Extracellular Brain
NK1.1 PerCp PK136 Biolegend 108726 1:800 Extracellular Brain
CD317 PerCp 927 Biolegend 127022 1:800 Extracellular Brain
Ly-6G PerCp 1A8 Biolegend 127654 1:800 Extracellular Brain
CD3 BV605 17A2 Biolegend 100237 1:400 Extracellular Spleen
CD4 BV785 RM4-5 Biolegend 100506 1:100 Extracellular Spleen
CD8a BV711 53-6.7 BD 563046 1:400 Extracellular Spleen
IFN-γ BV421 XMG1.2 Biolegend 505830 1:400 Intracellular Spleen
IL-17A PE-Cy7 eBio17B7 ThermoFisher 25-7177-82 1:400 Intracellular Spleen
IL-10 FITC JES5-16E3 Biolegend 505006 1:100 Intracellular Spleen
GM-CSF PE MP1-22E9 ThermoFisher 12-7331-82 1:100 Intracellular Spleen
CD45 FITC 30-F11 eBioscience 11-0451-85 1:400 Extracellular Brain
CD11b PE-Cy7 M1/70 eBioscience 25-0112-82 1:400 Extracellular Brain
Ly-6C PE HK1.4 eBioscience 12-5932-82 1:400 Extracellular Brain
FCRLS APC 4G11 Butovsky Lab 1:1000 Extracellular Brain
TCR-beta PerCp H57-597 Biolegend 109228 1:800 Extracellular Brain
B220 PerCp RA3-6B2 Invitrogen 45-0452-82 1:800 Extracellular Brain
NK1.1 PerCp PK136 Biolegend 108726 1:800 Extracellular Brain
CD317 PerCp 927 Biolegend 127022 1:800 Extracellular Brain
Ly-6G PerCp 1A8 Biolegend 127654 1:800 Extracellular Brain

Validation

Antibodies were titrated before use by our lab using primary mouse splenocytes stained with concentrations ranging between 1:100 and 1:1000 and tested by flow cytometry, the lowest concentration generating a good separation between populations was used for the experiments described in this study.

Animals and other research organisms

Policy information about studies involving animals; ARRIVE guidelines recommended for reporting animal research, and Sex and Gender in Research

Laboratory animals

Sex Genotype Treatment Age_start_of_treatment (y, mean and range) Age_sacrifice (y, mean and range)
Female APP/PS1-21 PBS (n=3) 2.63 (2-3.07) 4.95 (4.33-5.41)
Female APP/PS1-21 Bf (n=4) 2.45 (2-3.07) 5.02 (4.52-5.67)
Female Wildtype PBS (n=4) 2.63 (2-3.07) 4.84 (4.2-5.25)
Female Wildtype Bf (n=4) 2.68 (2-3.07) 5.11 (4.49-5.44)
Female APP/PS1-21 PBS (n=3) 2.73 (2.46-3.05) 5.49 (5.21-5.8)
Female APP/PS1-21 Bf (n=3) 2.63 (2.52-2.89) 5.39 (5.28-5.64)
Female Wildtype PBS (n=3) 2.69 (2.46-3.05) 5.45 (5.21-5.8)
Female Wildtype Bf (n=3) 2.73 (2.46-3.05) 5.48 (5.21-5.8)
Male APP/PS1-21 P BS (n=6) 2.79 (2.52-3.05) 5.55 (5.28-5.8)
Male APP/PS1-21 Bf (n=7) 2.79 (2.46-3.05) 5.55 (5.21-5.64)
Male Wildtype PBS (n=3) 3.04 (2.46-3.61) 5.79 (5.21-6.36)
Male Wildtype Bf (n=4) 3.1 (2.56-3.64) 5.85 (5.31-3.69)
Male Wildtype PBS (n=3) 12.6 (12.4-12.7) 15.0 (14.7-15.2)
Male Wildtype Bf (n=4) 12.5 (12.2-12.7) 14.9 (14.7-15.2)
Male Wildtype PBS (n=7) 8.6 (8.5-8.6) 11.56 (11.51-11.57)
Male Wildtype Bf (n=6) 8.4 (8.20-8.6) 11.35 (11.18-11.54)
Female Wildtype PBS (n=5) 11.8 (11.7-11.9) 14.2 (14.1-14.3)
Female Wildtype Bf (n=7) 11.8 (11.7-11.9) 14.2 (14.1-14.3)
Male APPS/PS1-dE9 PBS (n=5) 12.6 (12.4-12.7) 14.8 (14.5-14.9)
Male APPS/PS1-dE9 Bf (n=5) 12.5 (12.2-12.7) 14.7 (14.5-14.9)
Male APPS/PS1-dE9 PBS (n=3) 8.3 (7.5-8.6) 11.0 (10.5-11.3)
Male APPS/PS1-dE9 Bf (n=4) 8.4 (8.1-8.5) 11.2 (10.9-11.4)
Female Wildtype PBS (n=5) 14.0 (13.2-14.9) 16.0 (15.2-16.9)
Female Wildtype E3 (n=5) 14.0 (13.2-14.9) 16.0 (15.2-16.9)
Male Wildtype PBS (n=3) 13.5 (13.2-14.0) 15.5 (15.2-16.0)
Male Wildtype E3 (n=4) 13.8 (13.2-14.9) 15.8 (15.2-16.9)
Female 5xFAD H2O (n=8) 9.23 (6.67-11.13) 11.47 (8.95-13.34)
Female 5xFAD MTZ (n=9) 9.44 (6.67-11.13) 11.68 (8.95-13.34)
Female Wildtype H2O (n=7) 9.07 (8.2-9.67) 11.31 (10.46-11.9)
Female Wildtype MTZ (n=8) 9.34 (6.67-11.47) 11.53 (8.95-13.67)

The mice were kept on a 12 hour light/dark cycle, a temperature of 19-22°C and a humidity of 40-60%.

Wild animals

No wild animals were used.

Reporting on sex

We have tested the effects of B. fragilis in both female and male mice and observe similar results, although the effects of appear to be stronger in female APP/PS1 mice compared to males.

Field-collected samples

No field collected samples were used.

Ethics oversight

All animal experimental procedures were performed in accordance with the approved Animal Care and Use Protocols. The Institutional Animal Care and Use Committee (IACUC) at Harvard Medical School and Brigham and Women's Hospital has approved of all procedures involving animals.

Note that full information on the approval of the study protocol must also be provided in the manuscript.

Plants

Seed stocks

NA.

Novel plant genotypes

NA.

Authentication

NA.

Flow Cytometry

Plots

Confirm that:

☒ The axis labels state the marker and fluorochrome used (e.g. CD4-FITC).

☒ The axis scales are clearly visible. Include numbers along axes only for bottom left plot of group (a 'group' is an analysis of identical markers).

☒ All plots are contour plots with outliers or pseudocolor plots.

☒ A numerical value for number of cells or percentage (with statistics) is provided.

Methodology

Sample preparation

The mice were euthanized with CO2 until respiratory arrest and perfused before cardiac arrest by injecting 20 ml cold Hank's buffered saline solution (HBSS) buffer into the left ventricle of the heart. After perfusion, the mouse was decapitated and the brain and spleen were removed for fluorescence activated cell sorting (FACS).

The brain was homogenized, and immune cells were isolated by gradient centrifugation at 800xg over equal parts of 35% and 70% Percoll (GE Healthcare) at room temperature, the cells were collected from the middle layer. The cells were incubated with antibodies binding CD45-FITC, CD11b-PeCy7, Ly6C-PE and FCRLS-APC for 20 min at room temperature (Table 4). Cells were washed with FACS buffer (10% fetal bovine serum, 2.5% 4-(2-hydroxyethyl)-1-piperazineethanesulfonic acid (HEPES) and 0.4% ethylenediaminetetraacetic acid (EDTA) in HBSS), filtered through a 35µm cell strainer and mixed with 5µl 7-AAD live-dead stain (Becton, Dickinson and Company, BD, Franklin Lakes, NJ, USA). Microglia was sorted defined as CD45+ CD11b+ Ly6C-, and positive FCLS stain further distinguished microglia from peripherally recruited monocytes 52,53. 1000 microglia were sorted using an BD FACSAria II cell sorter (BD), and a dry pellet was stored at -80°C until RNA sequencing (see below).

The spleen was mashed through a 70 µm filter and red blood cells were lysed with ammonium-chloride-potassium (ACK) buffer. The cells were resuspended in complete IMDM medium (10% fetal calf serum (FBS), 1% penicillin-streptomycin, 0.1% β-mercaptoethanol, 1% non-essential amino acids) containing phorbol 12-myristate 13-aceate (PMA, 50 ng/ml, Sigma-Aldrich), ionomycin (1 µM, Sigma-Aldrich) and GolgiStop (1 µg/ml, BD) and incubated for 3h at 37°C and 5% CO2. After a wash in FACS buffer, the cell suspension was blocked for 20 min using anti-CD16/CD32 Fc-block (BD). Dead cells were stained with Aqua Zombie before fixation (in PBS, 1:1000, BioLegend). Next, the cells were incubated for 20 min at 4°C with antibodies targeting extracellular proteins. The antibodies were washed off and the cells were fixed for 20 min in Fixation/ Permeabilization buffer (eBioscience Foxp3 / Transcription Factor Staining Buffer Set, eBioscience, San Diego, CA, USA). The cells were washed with the Permeabilization buffer and then incubated with intracellular antibodies diluted with the same buffer. After a wash, cells were resuspended in FACS buffer and analyzed.

Instrument

BD LSRFortessa flow cytometer.

Software

FlowJo analysis software (BD).

Cell population abundance

Sorting strategy of microglia is described in figure 3B. We used the 4D4 antibody with high specificity for microglia to ensure the analyzed population did not contain peripheraly recruited monocytes, verified in Butovsky O Nat Neurosci. 2014. 1000 cells were used for RNA-seq analysis.

For T cell GM-CSF production analysis, the lowest recorded frequency was 0.41%, corresponding to 133 events.

Gating strategy

Microglia was defined as: CD11b+, CD45+, Ly6C-, 4D4+
GM-CSF producing T cells were defined as: CD3+, CD4+, GM-CSF+
Fluorescence minus one controls were used to make gates.

☒ Tick this box to confirm that a figure exemplifying the gating strategy is provided in the Supplementary Information.
